# Microalgae enhance cadmium accumulation in *Perilla frutescens*: root structural adaptation and secretion-mediated detoxification

**DOI:** 10.3389/fpls.2025.1642230

**Published:** 2025-09-02

**Authors:** Ying Ren, Yuying Su, Jinfeng Li, Hui Zhang, Yumeng Yang, Yuanyuan Li, Juan Chen, Xiaohui Pang, Zheng Zhang, Jianping Han

**Affiliations:** Institute of Medicinal Plant Development, Chinese Academy of Medical Sciences & Peking Union Medical College, Beijing, China

**Keywords:** *Perilla frutescens*, phytoremediation, microalgae, cadmium, root exudates

## Abstract

**Introduction:**

Cadmium (Cd) phytoremediation is often constrained by the low accumulation capacity and limited stress tolerance of most plant species. Developing approaches to overcome these limitations is essential for more efficient phytoremediation.

**Methods:**

The effect of microalgae supplementation on Cd phytoextraction in *Perilla frutescens* was evaluated under Cd stress, with plant growth, Cd accumulation, and root physiological, structural, and metabolic responses examined to elucidate the underlying mechanisms.

**Results:**

Microalgae supplementation significantly enhanced total plant biomass by 37.43% while increasing Cd accumulation by 20.72% in roots, 25.87% in stems, and 112.29% in leaves relative to Cd-only treatment. These improvements were closely associated with microalgae-induced physiological adaptations in roots, characterized by elevated peroxidase activity, reduced superoxide anion generation, and mitigated lipid peroxidation. Microalgae treatment also promoted Cd retention in root cell walls, with hemicellulose I showing a 166.37% increase in Cd binding (*p* < 0.05). Ultrastructural and spectroscopic analyses indicated that this enhancement likely stems from optimized root cell wall structure and increased functional group activity. Additionally, microalgae dramatically altered root exudate composition, increasing secretion of metal-chelating and antioxidant compounds such as ferulic acid, artemisinic acid, and dihydroartemisinin, whose concentrations were positively correlated with plant Cd accumulation.

**Discussion:**

These findings indicate that microalgae enhanced Cd phytoextraction in *Perilla frutescens* through modulation of root structural and metabolic traits, leading to enhanced Cd accumulation and tolerance. Microalgae-assisted phytoremediation may thus provide a viable strategy for remediating Cd-contaminated environments.

## 1 Introduction

Cadmium (Cd) contamination of soils has become a pressing environmental issue worldwide due to its persistence in ecosystems and potential threats to food safety (Li et al., 2023; Liang et al., 2024). Phytoremediation using hyperaccumulator plants presents an eco-friendly and cost-effective solution, yet its widespread application faces two key constraints: limited biomass production under heavy metal stress and inadequate metal accumulation efficiency in many species (Kafle et al., 2022; Li et al., 2023; Ren et al., 2024). *Perilla frutescens* has emerged as a promising candidate for Cd phytoremediation owing to its relatively high biomass and efficient Cd uptake (Reeves et al., 2018; Xiao et al., 2020). Importantly, Cd tends to accumulate in non-volatile forms in plants, minimizing contamination risks in essential oil extracts of *Perilla frutescens*, thereby enabling simultaneous phytoremediation and safe resource utilization (Xiao et al., 2020; Li Z. et al., 2024). However, maximizing its remediation potential requires a deeper understanding of the physiological mechanisms governing Cd uptake and tolerance.

Roots play a pivotal role in plant Cd sensing, uptake, and detoxification (Tao et al., 2020a). To cope with Cd stress, it has developed multiple adaptative strategies, among which cell wall sequestration is particularly important at the initial stage of metal entry (Thomas and Reid, 2021; Li et al., 2023). The Cd-binding capacity of the root cell wall is primarily determined by its polysaccharide components, particularly hemicellulose and pectin (Wei et al., 2023; Lin et al., 2024). For instance, in ramie, Cd bound to hemicellulose accounts for approximately 60% of the total Cd retained in the root cell wall (Ma et al., 2022). Likewise, Yu et al. (2020) reported that 59-65% of Cd in rice root cell walls was bound to pectin, and pectin removal reduced Cd binding by approximately 50%. Cd exposure typically induces the accumulation of these polysaccharide components and promotes their structural modifications such as pectin demethylesterification, which increases the number of negatively charged functional groups available for Cd binding (Guo et al., 2021; Hassan et al., 2024; Saeed et al., 2024). Such alterations enhance the Cd retention capacity of the cell wall and contribute to detoxification processes. Additionally, roots release a variety of metabolites such as organic acids and flavonoids that modulate Cd speciation and mobility in the rhizosphere (Meychik et al., 2021; Agarwal et al., 2024; Li et al., 2024; Xie et al., 2024). In *Sedum alfredii*, the secretion of low-molecular-weight organic acids such as tartaric and oxalic acids has been shown to facilitate Cd uptake by increasing its solubility and bioavailability (Tao et al., 2016; Tao et al., 2020b). These exudates also play a role in shaping microbial communities and nutrient dynamics, further supporting plant stress adaptation (Agarwal et al., 2024).

Conventional soil amendments have been explored to enhance phytoremediation but often come with inherent limitations. Synthetic chelators like EDTA can increase heavy metal bioavailability but have limitations such as poor biodegradability and potential phytotoxicity, restricting their field application (Hamidpour et al., 2024; Shi et al., 2024). In contrast, microalgae offer a sustainable biological alternative that addresses these constraints. Microalgae possess remarkable metal adsorption capabilities (Lee and Ryu, 2021; Manikandan et al., 2022; Yeheyo et al., 2024). For example, *Chlorella vulgaris* can remove up to 87% of Cd^2+^ from aqueous environments within 18 days (Al-Khiat et al., 2023). Other species like *C. pyrenoidosa*, *Parachlorella kessleri*, and *Tolypothrix tenuis* also showed potential in heavy metal remediation (Nagase et al., 2005; Bauenova et al., 2021; Manikandan et al., 2022; Ran et al., 2024). Beyond direct metal removal, microalgae function as effective biostimulants and soil amendments (Lee and Ryu, 2021; Moon et al., 2024). Their photosynthetic activity supplies oxygen and organic matter to support microbial and plant activity. Moreover, the secreted compounds of microalgae has been reported to induce physiological and structural changes in plants that protect them against biotic and abiotic stresses and promote their growth (El Arroussi et al., 2018; Carillo et al., 2020; Lee and Ryu, 2021). Microalgae can also facilitate the mineralization and solubilization of primary macro- and micronutrients in the soil and improve soil conditions (Cao et al., 2023; Lorentz et al., 2024). Notably, their high environmental adaptability allows them to thrive not only in aquatic environments but also in plant tissues and soil (Lee and Ryu, 2021). Compared to single strains, microalgae consortia offer greater functional stability and synergistic benefits, making them more suitable for application under complex field conditions (Mattsson et al., 2021; Díaz et al., 2024; Mollo et al., 2024; Singh and Singh, 2024).

While microalgae have shown promise in phytoremediation and plant growth promotion, their regulatory roles in plant Cd uptake and tolerance remain poorly understood. Therefore, employing a standardized microalgae consortium, this study focused on how microalgae supplementation influences Cd accumulation in *Perilla frutescens*, with particular focus on root-level adaptations. A hydroponic system was used to minimize environmental complexity and provide a controlled setting to isolate the direct effects of microalgae on root Cd uptake and stress responses. By examining changes in root ultrastructure, cell wall composition, antioxidant responses, and exudate characteristics, this research sought to unravel the mechanism basis of microalgae-assisted phytoremediation. The findings are expected to maximize plant productivity and ecological safety and support the development of sustainable strategies for remediating Cd-contaminated soils.

## 2 Materials and methods

### 2.1 Experimental design

*Perilla frutescens* seeds were sterilized with 0.5% NaClO solution for 10 min, and rinsed with deionized water. The seeds were then sown in soil and covered with a thin layer of peat soil for natural germination. After 50 days, when the seedlings reached approximately 10 cm in height, vigorous and uniformly sized seedlings were transplanted into polyethylene plastic containers (310×285×180 mm). The seedlings were grown in a controlled growth chamber set at 25 °C with a 16 h light/8 h dark photoperiod. Initially, the seedlings were precultured in Hoagland’s modified nutrient solution (Beijing Coolabor Technology Co., Ltd., Beijing) with concentration gradients from 1/4 to full strength (the composition of the Hoagland’s modified nutrient solution is provided in **Supplementary Table S1**). After 10 days of acclimation, the seedlings were transferred into Hoagland’s modified nutrient solution with different treatments as designed. Cd stress was applied using CdCl_2_·2.5H_2_O (98% purity, Shanghai Macklin Biochemical Co., Ltd.), at a concentration of 1 mg/L, which was selected based on the study by Tang et al. (2025) and Vasudhevan et al. (2025) for its effectiveness in inducing measurable Cd stress responses in plants. Three treatments were designated: CK (no Cd stress), ST (1 mg/L Cd treatment), and SM (1 mg/L Cd treatment with microalgae addition). Each treatment included three biological replicates, with six seedlings per replicate. The microalgae additive used was obtained from Beijing Bailing Biotechnology Co., Ltd., consisting mainly of *Chlorella pyrenoidosa*, *Anabaena azotica*, and *Tolypothrix tenuis*, with a concentration exceeding 1×10^6^ cells/mL, applied at a 100-fold dilution. During cultivation, the nutrient solution was aerated daily using an oxygen pump and refreshed every three days. Plant growth parameters (height, stem diameter, and biomass) were monitored regularly. After 24 days of transplantation, plants were harvested for analysis.

### 2.2 Collection and extraction of root exudates

After harvesting, healthy and uniform *Perilla frutescens* seedlings were selected for subsequent experiments. The roots were rinsed with 5 mmol/L Na_2_EDTA and deionized water (2–3 times each) to ensure complete removal of residual Cd^2+^ and nutrients. To specifically collect plant-derived root exudates without interference from other organisms, the cleaned seedlings were transferred to a sterile flask containing sterile water with roots fully submerged, and the flask was wrapped in aluminum foil to block light. In a 25 °C light incubator, root exudates were collected for 6 h. The collected liquid was filtered using double-layered filter paper, concentrated, and then subjected to further filtration through a 0.22 μm filter membrane. Finally, the processed liquid was rapidly frozen in liquid nitrogen for 5 min and stored at -80 °C.

Samples were extracted using 80% methanol. A 100 μL aliquot of the sample was re-suspended in 400 μL of the 80% methanol. After incubating the mixture on ice for 5 min, it was centrifuged at 15,000 g and 4 °C for 20 min. The supernatant was then diluted to final concentration containing 53% methanol and the centrifugation step was repeated. The final supernatant was injected into the LC-MS/MS system for analysis.

### 2.3 Untargeted metabolomics analysis and data processing

The root exudates were analyzed using untargeted metabolomics assay from Novogene Co., Ltd. (Beijing, China). UHPLC-MS/MS analysis was carried out on a Vanquish UHPLC system coupled to a Q Exactive™ HF-X mass spectrometer (Thermo Fisher Scientific, Germany). A Hypersil Gold column (100×2.1 mm, 1.9 μm) was employed to separate root exudates using a 12-minute linear gradient. The column temperature was maintained at 40 °C, with a flow rate of 0.2 mL/min. The mobile phases for the positive and negative polarity modes consisted of 0.1% formic acid (A) and methanol (B), with the following elution gradient: 0-1.5 min, 98% A; 1.5–3 min, 98-15% A; 3–10 min, 15-0% A; 10-10.1 min, 0-98% A; 10.1–12 min, 98% A. The Q Exactive™ HF-X mass spectrometer was operated in positive/negative polarity mode, with specific parameters detailed in **Supplementary Table S2**.

Quality control (QC) samples, prepared by pooling equal volumes of all experimental samples, were used to equilibrate the LC-MS system and monitor instrument performance throughout the run. Blank samples were included to eliminate background ion interference during data processing. Since all samples were randomized and analyzed within a single batch, batch effects were considered minimal. Raw mass spectrometry data were processed using Compound Discoverer 3.3 (ThermoFisher), encompassing peak alignment, picking, and area quantification. Data processing parameters included an actual mass tolerance of 5 ppm, signal intensity tolerance of 30%, and peak areas were normalized against the first quality control (QC) sample. Minimum intensity thresholds were applied to filter noise. Following initial processing, peak intensities were normalized to total spectral intensity. Molecular formula prediction was performed based on analysis of additive ions, molecular ion peaks, and fragment ions. Metabolite identification was based on spectral matching against the mzCloud (https://www.mzcloud.org/), mzVault, and MassList databases. Statistical analyses were performed using CentOS (CentOS release 6.6), R (version 3.4.3) and Python (version 2.7.6). Data were subsequently scaled and standardized for statistical analysis. Quality control measures included removal of compounds showing coefficient of variation (CV) greater than 30% in QC samples.

Functional annotation was performed using the KEGG (https://www.genome.jp/kegg/pathway.html) and HMDB (https://hmdb.ca/metabolites) databases. Principal component analysis (PCA) and partial least squares-discriminant analysis (PLS-DA) were conducted with metaX. Differentially accumulated metabolites (DAMs) were identified based on variable importance in projection (VIP) > 1, p-value< 0.05 (t-test), and fold change (FC) ≥ 2 or ≤ 0.5. Volcano plots, clustering heatmaps, Mantel correlation analyses, and random forest analyses were generated in R using standard packages. Z-score transformation was applied for clustering analysis, and metabolite-metabolite correlations were assessed using Pearson correlation coefficients. Functional enrichment analysis of DAMs was carried out based on KEGG pathway classification.

### 2.4 Transmission electron microscopy analysis of *Perilla frutescens* root apex

The *Perilla frutescens* root samples were initially washed with 5 mmol/L Na_2_EDTA and deionized water. Root apex, approximately 1 cm in length, were then excised and placed in a 2.5% glutaraldehyde solution for fixation at 4 °C for 12 h. Following this, the samples were rinsed three times with 0.1 mol/L phosphate buffer (pH 7.2), with each rinse lasting 15 min. Subsequently, the samples were immersed in a solution containing 1% osmium tetroxide and 0.1 mol/L phosphate buffer (pH 7.2) at room temperature for 2 h, followed by three additional rinses with the same buffer. Dehydration was carried out using a graded ethanol series at concentrations of 30%, 50%, 70%, 80%, 85%, 90%, 95%, and 100%, each step lasting 15–20 min. They were then infiltrated with a series of acetone and epoxy resin mixtures at 37 °C, using the following ratios: acetone-resin (2:1), acetone-resin (1:1), and pure epoxy resin, each for 8–12 h. After embedding, the samples were sectioned to a thickness of 80–100 nm, stained, and examined using transmission electron microscopy (TEM) (HT7700, Hitachi, Japan).

### 2.5 Evaluation of Cd content in different tissues

The quantification of Cd was conducted through inductively coupled plasma-mass spectrometry (ICP-MS), following the guidelines of the National standard of the People’s Republic of China (GB 5009.15—2014). *Perilla frutescens* plants were divided into roots, stems, and leaves, then dried, ground and sieved. The resulting powder was digested overnight in a covered container with a nitric acid-perchloric acid mixture (*v*/*v*, 9:1). The mixture was then heated on a heating plate until white fumes appeared and the solution turned colorless or pale yellow (if the solution turned brown during heating, additional acid mixture was added). After cooling, the digestion solution was diluted to 10 mL with 2% nitric acid and filtered through 0.45 μm membrane filters. The Cd concentration in the samples was then measured using an ICP-MS (iCAP Qc, Thermo Fisher, Germany). Instrument parameters are detailed in **Supplementary Table S3**. The translocation factor (TF), which assesses the efficiency of Cd translocation between different plant tissues, was calculated using the following formulas (Hussain et al., 2024):


$${\text{TF}}_{root-to-stem}=\frac{Cd\;concentration\;in\;stem\text{ (mg}/\text{kg DW)}}{Cd\;concentration\;in\;root\text{ (mg}/\text{kg DW)}}$$

$${\text{TF}}_{root-to-leaf}=\frac{Cd\;concentration\;in\;leaf\text{ (mg}/\text{kg DW)}}{Cd\;concentration\;in\;root\text{ (mg}/\text{kg DW)}}$$

$${\text{TF}}_{stem-to-leaf}=\frac{Cd\;concentration\;in\;leaf\text{ (mg/kg DW)}}{Cd\;concentration\;in\;stem\text{ (mg/kg DW)}}$$

### 2.6 Cd content measurement in root cell walls

The separation of subcellular component from *Perilla frutescens* roots was modified based on the method described by Wei et al. (2021). Approximately 1 g of fresh root sample was homogenized with 10 mL of pre-cooled extraction buffer (250 mmol/L sucrose, 50 mmol/L Tris-HCl (pH 7.5), 1.0 mmol/L dithiothreitol, and 5 mmol/L ascorbic acid). The homogenate was then centrifuged at 2000 rpm for 15 min at 4 °C to collect the precipitate as the cell wall fraction. The centrifugation steps were repeated twice. The resulting cell wall fraction was freeze-dried at -80 °C to constant weight. The Cd concentration in cell wall samples was determined following the procedure outlined in section 2.5.

### 2.7 Extraction of root cell wall and its polysaccharide components

The extraction method of root cell walls was adapted from Huang et al. (2023). Fresh *Perilla frutescens* root samples (~1.5 g) were washed with deionized water and ground into a fine powder using liquid nitrogen. The powder was then suspended in 10 mL of 75% ethanol pre-cooled to 4 °C and incubated in an ice bath for 20 min. After incubation, the mixture was centrifuged at 4 °C, 8000 rpm for 10 min. The resulting precipitate was subsequently washed three times with 10 mL each of pre-cooled acetone, a methanol-chloroform mixture (1:1, *v*/*v*), and methanol. Each wash step involved incubation in an ice bath for 20 min and centrifugation at 4 °C, 8000 rpm for 10 min. The final precipitate was rinsed twice with deionized water pre-cooled to 4 °C, freeze-dried, and stored at 4 °C for further analysis.

Polysaccharides from root cell walls were extracted following the protocols of Wang (2022) and Wei et al. (2023). Approximately 5 mg of cell wall material was incubated in 1 mL of 0.5% ammonium oxalate buffer (containing 0.1% NaBH_4_) at 100 °C for 1 h, and then centrifuged at 12000 rpm for 10 min, yielding pectin in the supernatant. This process was repeated three times, and the supernatants were pooled. The residue was then treated with 1 mL of 4% KOH (containing 0.1% NaBH_4_) at room temperature for 12 h. Following centrifugation at 12000 rpm for 10 min, the supernatants, collected twice, constituted hemicellulose I. The precipitate was washed with deionized water and extracted with 1 mL of 24% KOH (containing 0.1% NaBH_4_) using the same conditions to obtain hemicellulose II. The remaining pellet, identified as cellulose, was washed with deionized water and dissolved in 1 mL of 72% H_2_SO_4_ (Dang et al., 2023). After cooling in an ice bath for 30 min, the solution was filtered through a 0.22 μm filter membrane.

### 2.8 Fourier transform infrared spectroscopy analysis

Approximately 2 mg of dried root cell wall powder was thoroughly mixed with 100 mg of KBr and ground uniformly. The mixture was then pressed into a tablet under a pressure of 20 MPa. A fourier transform infrared (FTIR) spectrometer (Nicolet 6700, Thermo Fisher Scientific, USA) was used for spectral analysis. The wavelength range for detection was 400–4000 cm^-1^, with a resolution of 4 cm^-1^ and a signal-to-noise ratio of 50000:1. Each sample was scanned 32 times.

### 2.9 Quantification of polysaccharide content in root cell walls and Cd concentration in polysaccharide components

The pectin content in the root cell walls was quantified by measuring the galacturonic acid content, following the method described by Blumenkrantz and Asboe-Hansen (1973). Specifically, 200 μL of pectin extract was reacted with 1 mL of concentrated sulfuric acid (containing 0.0125 mol/L sodium tetraborate) at 100 °C for 5 min. After cooling to room temperature, 20 μL of 0.15% meta-hydroxydiphenyl (*w*/*w*) was added, and the mixture was allowed to react for 20 min. The absorbance was then measured at 520 nm. The galacturonic acid (Shanghai yuanye Biotechnology Co., Ltd, China) (purity ≥ 98%) was used as a reference standard.

The hemicellulose content was determined by quantifying total sugars according to the method of DuBois et al. (1956) with modifications. For this, 1 mL of hemicellulose I and hemicellulose II extracts, which had been two-fold diluted with distilled water, were incubated with 800 μL of 5% phenol (*w*/*w*) and 5 mL of concentrated sulfuric acid at room temperature for 15 min, followed by incubation at 100 °C for 15 min. Once cooled, the absorbance was measured at 490 nm.

The cellulose content was assessed using the anthrone colorimetric method (Griffiths et al., 2014). The cellulose filtrate was diluted tenfold, and 200 μL of the diluted filtrate was taken. Then, 50 μL of ethyl acetate contain 2% anthrone solution (*w*/*w*) and 500 μL of concentrated sulfuric acid were added. The solution was mixed thoroughly, incubated in boiling water for 10 min, then cooled. The absorbance was measured at 620 nm. The D-glucose (Shanghai Yuanye Biotechnology Co., Ltd., China) (purity ≥ 98%) served as the reference standard for hemicellulose and cellulose quantification.

The Cd concentration in the aforementioned components was quantified using ICP-MS, as described in section 2.5.

### 2.10 Assay of root antioxidant activity

The contents and generation rates of superoxide anion radicals, the malondialdehyde (MDA) content, and the activities of superoxide dismutase (SOD) and peroxidase (POD) in root samples were determined using biochemical kits. Detailed information on the kits is provided in **Supplementary Table S4**.

### 2.11 Statistical analysis

One-way analysis of variance (ANOVA) was performed using IBM SPSS Statistics 23.0 to analyze variance. Means were compared using Tukey’s HSD test (for data meeting homogeneity of variance) and Tamhane’s T2 test (for data not meeting homogeneity of variance), with a significance threshold of 0.05. Graphs were plotted using GraphPad Prism (version 8.0.2), R (version 4.3.0), and Origin 2024b (version 10.1.5.132).

## 3 Result and discussion

### 3.1 Growth conditions

**Figure 1:**
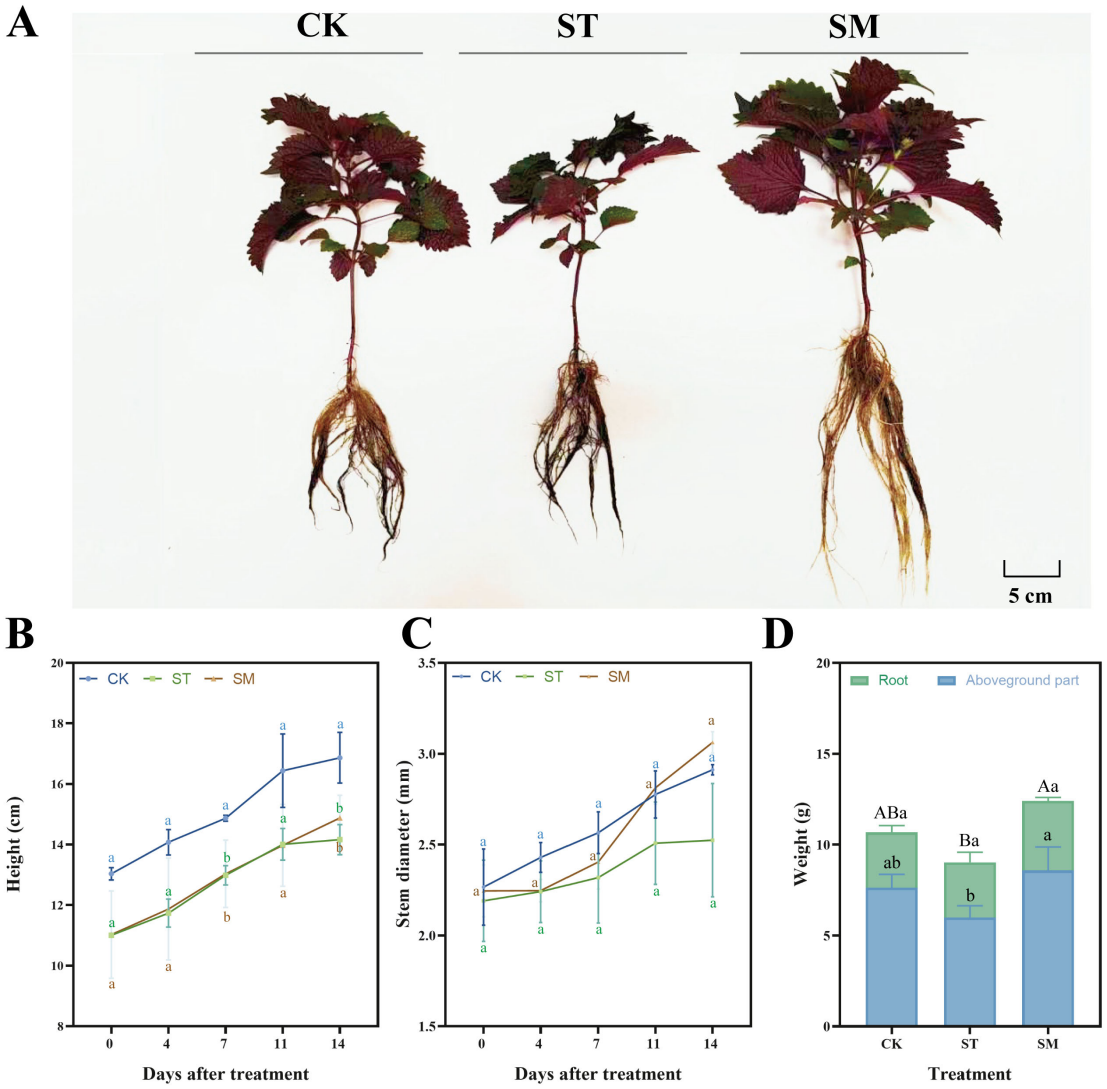
Growth performance of *Perilla frutescens* under different treatments. **(A)** Plant morphology. **(B)** Dynamic changes in plant height during the treatment period. **(C)** Dynamic changes in stem diameter during the treatment period. **(D)** Fresh biomass at harvest. Note: The values are presented as mean ± SD (standard deviation). The different lowercase letters in panels B, C, and D indicate significant differences among treatment groups at each time point (*p<*0.05). The different uppercase letters in panel D indicate significant differences in total biomass among treatment groups at *p<*0.05.

The growth dynamics of *Perilla frutescens* were monitored throughout the experimental period, with measurements taken for plant height, stem diameter, and fresh weight of the aboveground parts and roots at harvest. The results demonstrated that Cd stress suppressed plant growth, leading to reduced plant height, stem diameter, and plant biomass compared to the control group (**Figures 1B-D**). In contrast, the introduction of microalgae significantly enhanced the aboveground biomass (43.20%) and total biomass (37.43%), and also promoted increases in plant height and stem diameter. Morphologically, plants treated with microalgae exhibited a more robust appearance, with better-developed root systems, thicker stems, and larger leaf area (**Figure 1A**). Besides, the microalgae treatment accelerated the growth rate of *Perilla frutescens*, indicating its potential to mitigate the adverse effects of Cd stress.

Cd stress typically exerts detrimental effects on plant growth, manifesting as attenuated growth performance like inhibited height, stem diameter, and biomass accumulation, as observed in this study. These growth impairments are closely related to Cd-induced oxidative stress, disruption of essential nutrient uptake, impaired photosynthetic efficiency, and altered metabolic activities (Genchi et al., 2020; Hassan et al., 2024). Specifically, the slowed growth and compromised plant vitality under Cd stress reflect the severe physiological disturbances caused by heavy metal toxicity, which interfered with cellular processes and led to stunted development.

Microalgae, particularly *Chlorella* and *Scenedesmus* species, are recognized beneficial biofertilizers (Dineshkumar et al., 2019; Suchithra et al., 2022; Cao et al., 2023; Eckstien et al., 2024). They are capable of providing essential nutrient and oxygen for plant growth through processes such as photosynthesis, nitrogen fixation and phosphorus solubilization (Renuka et al., 2018; Renganathan et al., 2024). For instance, the application of microalgae biomass, predominantly *Scenedesmus* sp., resulted in increased organic matter, nitrogen and phosphorus levels (Castro et al., 2024). Treatment with *Chlorella vulgaris* and *Spirulina platensis* significantly enhanced maize growth performance (Dineshkumar et al., 2019). The bioactive compounds secreted by microalgae, such as polysaccharides and phytohormones, likely play an important role in enhancing stress tolerance as well (Yehuda et al., 2022; León-Vaz et al., 2023). El Arroussi et al. (2018) found that microalgae polysaccharides could improve the growth of tomatoes under salt stress, increasing chlorophyll contents and promoting protein accumulation. These exudates can mitigate oxidative damage, modulate plant growth pathways, and strengthen cellular defenses, thereby allowing plants to maintain more stable growth under stress conditions (Eckstien et al., 2024; Renganathan et al., 2024). In addition, microalgae have been shown to adsorb Cd ions, which could reduce free Cd concentrations in the rhizosphere, potentially creating a less toxic environment for plant development (Al-Khiat et al., 2023; Ali et al., 2024). While these aspects were not directly tested in our study, the observed improvements in plant growth metrics in the presence of microalgae suggest that microalgae serve as an effective plant growth promoter and can enhance stress tolerance in plants exposed to Cd. On the other hand, the faster growth rate and greater biomass accumulation seen in microalgae-treated plants are also critical from a phytoremediation perspective. Plants that grow faster, produce more biomass, and develop more robust root systems are better equipped to absorb and sequester heavy metals from the environment, making microalgae a valuable ally in improving growth and heavy metal removal efficiency of hyperaccumulators in contaminated environments.

### 3.2 Physiological responses of *Perilla frutescens* roots to Cd stress

Cd stress induced pronounced oxidative damage in *Perilla frutescens* roots, as evidenced by a 17.69% increase in both the content and production rate of superoxide anion radicals, and an 88.10% increase in malondialdehyde (MDA) levels compared to the control (**Supplementary Figure S1A, B, E**). In response, the antioxidant enzyme was activated, showing 40.88% and 142.85% increases in superoxide dismutase (SOD) and peroxidase (POD) activities, respectively (**Supplementary Figure S1C, D**). Notably, microalgae supplementation altered this response pattern. The POD activity further rose by 27.98%, while superoxide anion radical content and its production rate, as well as MDA content all decreased compared to Cd stress alone. Nevertheless, the SOD activity under microalgae treatment was significantly lower than that in Cd-only group.

The antioxidant defense system plays a central role in plant adaptation to heavy metal stress, particularly through maintaining reactive oxygen species (ROS) homeostasis (Hasanuzzaman et al., 2020). Plants face a critical challenge when exposed to Cd stress, primarily due to the surge in ROS that overwhelms their cellular defense mechanisms (Sharma et al., 2022). This oxidative burst, characterized by the accumulation of various ROS, including superoxide anions and hydrogen peroxide (H_2_O_2_), can compromise cell membranes and damage essential biomolecules (Hasanuzzaman et al., 2020; Wang P. et al., 2024). Lipid peroxidation, reflected by elevated MDA content, and the accumulation of superoxide anions observed here highlighted the severity of oxidative damage. The sharp rise in SOD and POD activities reflected the plant’s attempt to cope with this stress, yet the persistence of oxidative markers implies that endogenous defenses were not fully effective. The addition of microalgae offered a relief, as demonstrated by the reductions in both superoxide anion levels and MDA contents. This aligns with established knowledge about microalgal capabilities. Microalgae are known to secrete a variety of bioactive compounds, such as phenolics, amino acids, and organic acids, which have been shown to modulate oxidative stress and support plant recovery (Parmar et al., 2023; Čmiková et al., 2024). While the present study did not characterize these secretions, the physiological outcomes—reduced oxidative damage—imply their possible involvement.

What stands out in this study is the differential response of the antioxidant enzymes. While POD activity continued to rise following microalgae treatment, likely improving H_2_O_2_ detoxification, SOD activity dropped. This pattern is consistent with findings from previous studies. For example, exopolysaccharides from *Dunaliella salina* attenuated the drought-induced elevation of SOD activity in tomato (El Arroussi et al., 2018). Similarly, *Aphanothece* crude extracts significantly suppressed SOD activity in Cd-stressed tomato plants, while algal extracts enhanced POD activity in *Brassica chinensis* under Cd exposure (Fal et al., 2023; Shaari et al., 2023). These findings reflect distinct interactions between microalgae and plant oxidative responses. The differential enzyme activities observed in present study may be attributed to the action of microalgae-derived compounds such as exopolysaccharides, organic acids, and antioxidant peptides, which are known to chelate Cd ions or directly scavenge superoxide anions, thereby reducing the superoxide anions accumulation and diminishing the demand for SOD-mediated detoxification (Gitau et al., 2023; Guehaz et al., 2023; Parmar et al., 2023; Xiao et al., 2023). Additionally, since H_2_O_2_ is a potentially harmful byproduct of SOD activity, its excessive accumulation may also contribute to SOD inactivation. In parallel, the continued rise in POD activity likely reflects an adaptive response to elevated H_2_O_2_ or cell wall remodeling processes such as lignification, which are commonly activated in plant defense against heavy metal stress. Collectively, such results suggested that microalgae may modulate the antioxidant response strategy of *Perilla frutescens* under Cd stress, altering the plant resistance on specific antioxidant pathways and reducing the demand for SOD-driven detoxification.

### 3.3 Cd accumulation in different tissues of *Perilla frutescens*

**Figure 2:**
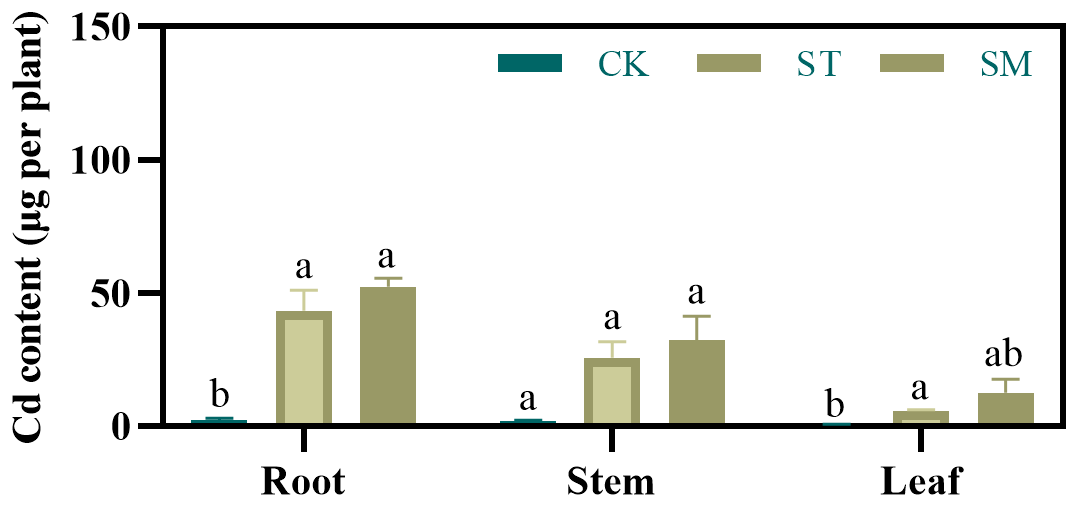
Effect of different treatments on Cd contents of *Perilla frutescens* roots, stems, and leaves. Note: The values are presented as mean ± SD (standard deviation). The different lowercase letters indicate significant differences at *p<*0.05.

Compared to the control group, Cd concentrations in the roots, stems, and leaves were significantly increased under Cd treatment (**Supplementary Figure S2**). Microalgae application further enhanced Cd accumulation, with Cd concentrations in stem and leaf rising by 17.09% and 56.66%, respectively, compared to Cd treatment alone. Moreover, relative to the ST group, microalgae amendment raised Cd content in the roots, stems, and leaves by 20.72%, 25.87%, and 112.29%, respectively (**Figure 2**). Also, microalgae increased translocation factor (TF) values by 7.46% for root-to-stem translocation, 53.71% for root-to-leaf translocation, and 22.28% for stem-to-leaf translocation (**Supplementary Table S5**).

The observed enhancement in Cd accumulation suggested that microalgae can effectively improve phytoextraction efficiency. This enhancement is likely driven by microalgae-induced reduction of oxidative stress and promotion of plant growth. However, considering the differences in alterations in Cd concentrations and contents observed prior to and following microalgae treatment, it appears that the increased Cd load in *Perilla frutescens* may be more attributable to biomass augmentation.

### 3.4 Root apical cell ultrastructure analysis via transmission electron microscopy

**Figure 3:**
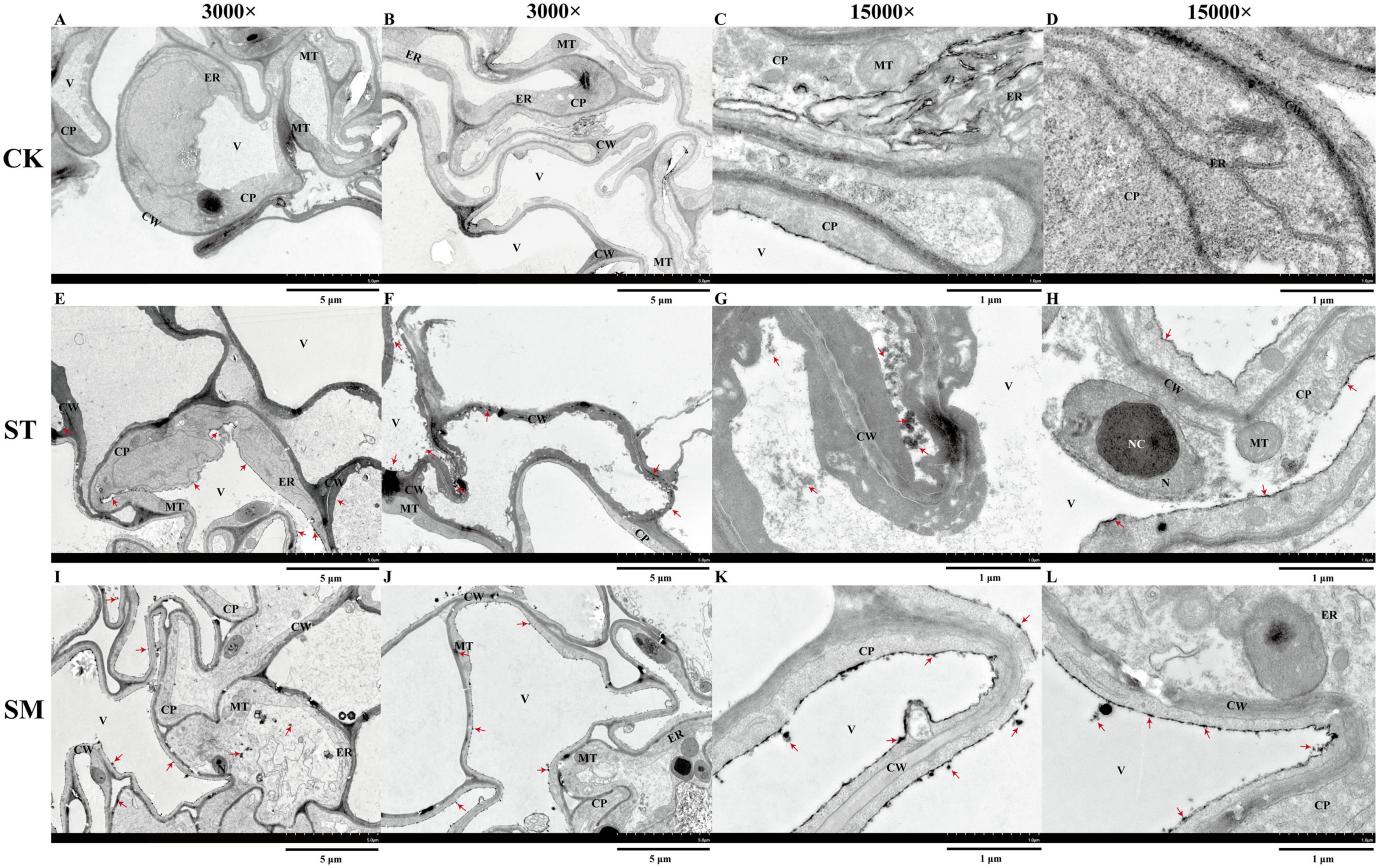
Ultrastructure of root apical cells under different treatments observed via transmission electron microscopy (TEM). **(A, B)** Control group (CK) at 3,000×. **(C, D)** CK group at 15,000×. **(E, F)** Cd-treated group (ST) at 3,000×. **(G, H)** ST group at 15,000×. **(I, J)** Cd and microalgae co-treatment group (SM) at 3,000×. (K, L) SM group at 15,000×. Note:CW, cell wall; V, vacuole; ER, endoplasmic reticulum; MT, mitochondria; CP, cytoplasm; NC, nucleolus; N, nucleus. Red arrows point to dark deposits that may represent Cd-chelated compounds.

To evaluate the impact of Cd stress and microalgae treatment on the ultrastructure of root cells, transmission electron microscopy (TEM) was employed for observation of root apical tissues. Root apical cells in the control group displayed intact structures, with uniformly thin cell walls and mitochondria showing a normal and rounded appearance (**Figures 3A-D**). However, Cd exposure induced marked subcellular damage. The nuclear membrane appeared disrupted, and nucleoli were enlarged (**Figure 3H**), which may be associated with the enhanced genetic regulation and cellular responses aimed at coping with Cd toxicity. The thickening of the cell walls, likely a defensive adaptation, was also observed, along with damage to membrane structures (**Figures 3E, F**). The observed damage to the tonoplast suggested possible Cd shift from organelles into the cytoplasm (**Figures 3E-H**). Besides, the dark deposits, presumably representing Cd accumulation, were visible in the cell walls, vacuoles and cytoplasm. Following microalgae treatment, several indicators of improved cellular stress resistance were observed, including restored membrane integrity and increased numbers of mitochondria and endoplasmic reticulum, potentially reflecting increased metabolic activity to support recovery from Cd stress (**Figures 3I-L**). Although cell walls remained thickened compared to the control group, this persistent thickening may indicate a long-term protective response reinforced by microalgae treatment. Moreover, the increased presence of dark deposits in root apical cells may suggest further Cd accumulation, implying that microalgae potentially enhance the plant ability to sequester and tolerate higher levels of Cd.

### 3.5 Cd concentration in root cell walls of *Perilla frutescens*

This study revealed that Cd adsorption in root cell wall of *Perilla frutescens* was significantly enhanced upon Cd exposure, with further amplification by microalgae treatment (**Supplementary Figure S3**). The cell wall serves as the first barrier to heavy metals, and its components—primarily polysaccharides and structural proteins—contain a variety of functional groups such as carboxyl, hydroxyl, and amino groups capable of binding Cd via coordination or electrostatic interactions (Wei et al., 2023; Li Z. et al., 2024; Xie et al., 2024). These interactions reduce the content of free Cd in the cytoplasm and facilitate metal detoxification (Meychik et al., 2021; Hassan et al., 2024). The enhanced Cd retention in the root cell wall under microalgae treatment points to a more active sequestration process at this structural barrier, which likely contributes to the increased Cd accumulation observed in roots (Zhao et al., 2021; Wei et al., 2023). Differences in cell wall composition and structure across plant species can account for their differing capacities to accumulate Cd (Guo et al., 2021). While increased Cd retention in the cell wall generally correlates with restricted translocation and reduced cellular damage, the current study also recorded enhanced Cd translocation to aerial tissues in microalgae-treated plants, an observation that appears counterintuitive relative to commonly accepted mechanisms (Lu et al., 2020; Saeed et al., 2024). Interestingly, a similar trend was reported by Li Z. et al. (2024), who found that application of 300-times diluted bamboo vinegar under Cd stress promoted both Cd chelation in roots and its upward transport in *Perilla frutescens*. Several factors may contribute to this phenomenon. Firstly, microalgae addition alleviated oxidative stress in *Perilla frutescens*, as evidenced by the reduced levels of superoxide anion radicals. This reduction in oxidative damage likely improved plant physiological status, thereby enhancing its capacity for Cd uptake and redistribution. Secondly, microalgae are known to secrete a variety of bioactive compounds, including phytohormones, flavonoids, and organic acids, which can alter the chemical speciation and mobility of Cd in the rhizosphere (Guehaz et al., 2023; Seregin and Kozhevnikova, 2024; Wang J. et al., 2024). These compounds may increase Cd bioavailability by promoting its solubilization through chelation or rhizosphere acidification (Tao et al., 2020b; Xie et al., 2024). The enhanced Cd uptake under improved root vitality could subsequently promote greater translocation of Cd to shoots. It is also plausible that microalgae treatment may modulate the expression or activity of Cd transporters involved in long-distance translocation, although this possibility remains untested in the current study. Another possible explanation is that once the available Cd-binding sites in the cell wall become saturated, excess Cd may be redirected to the aerial parts to avoid toxic accumulation in the roots. Nonetheless, these mechanisms remain hypothetical and require further experimental validation.

### 3.6 Cd binding and compositional changes of root cell wall polysaccharides

Cd stress induced alterations in the composition of root cell wall polysaccharides in *Perilla frutescens*. Compared to the control, the levels of pectin and hemicellulose II increased by 13.92% and 6.41%, respectively, while hemicellulose I and cellulose contents decreased by 15.89% and 16.50% (**Supplementary Figure S4A-D**). Although microalgae treatment did not cause statistically significant changes in the abundance of these polysaccharide components, it elevated the Cd concentration bound to hemicellulose I by 166.37% (*p*<0.05), suggesting a substantial enhancement in the Cd-binding capacity of this fraction (**Supplementary Figure S5**).

Polysaccharides are integral components of the plant cell wall, maintaining structural integrity and modulating mechanical properties (Wei et al., 2023). Through their rich negatively charged functional groups, these polysaccharides can effectively chelate metal cations, thereby alleviating heavy metal toxicity (Guo et al., 2021; Yu et al., 2021). Pectin and hemicellulose are recognized as the primary Cd-binding constituents, as studies revealed that their removal significantly reduced Cd adsorption (Yu et al., 2021; Wei et al., 2023). Similarly, enhanced hemicellulose content through genetic modification was shown to promote Cd accumulation in cell walls (Ma et al., 2024). Although a positive correlation between polysaccharide abundance and metal retention is well-documented (Wu et al., 2021; Ma et al., 2024), the present study revealed that the remarkable increase in Cd bound to hemicellulose I following microalgae treatment occurred without a corresponding rise in its content. This is consistent with findings reported by Li Z. et al. (2024), who likewise observed that enhanced Cd retention in hemicellulose I was not driven by the increase in its quantity. While the underlying mechanism remains unclear, the enhanced Cd retention may result from microalgae-induced structural changes in hemicellulose I, potentially including chemical modification of functional groups and changes in its branching or cross-linking patterns. Such alternations may increase the accessibility or density of active binding sites, thereby improving its metal chelation efficiency (Liu et al., 2020). However, further investigation is required to clarify the mechanisms underlying microalgae-driven Cd accumulation in hemicellulose.

### 3.7 FTIR analysis of *Perilla frutescens* root cell walls

**Figure 4:**
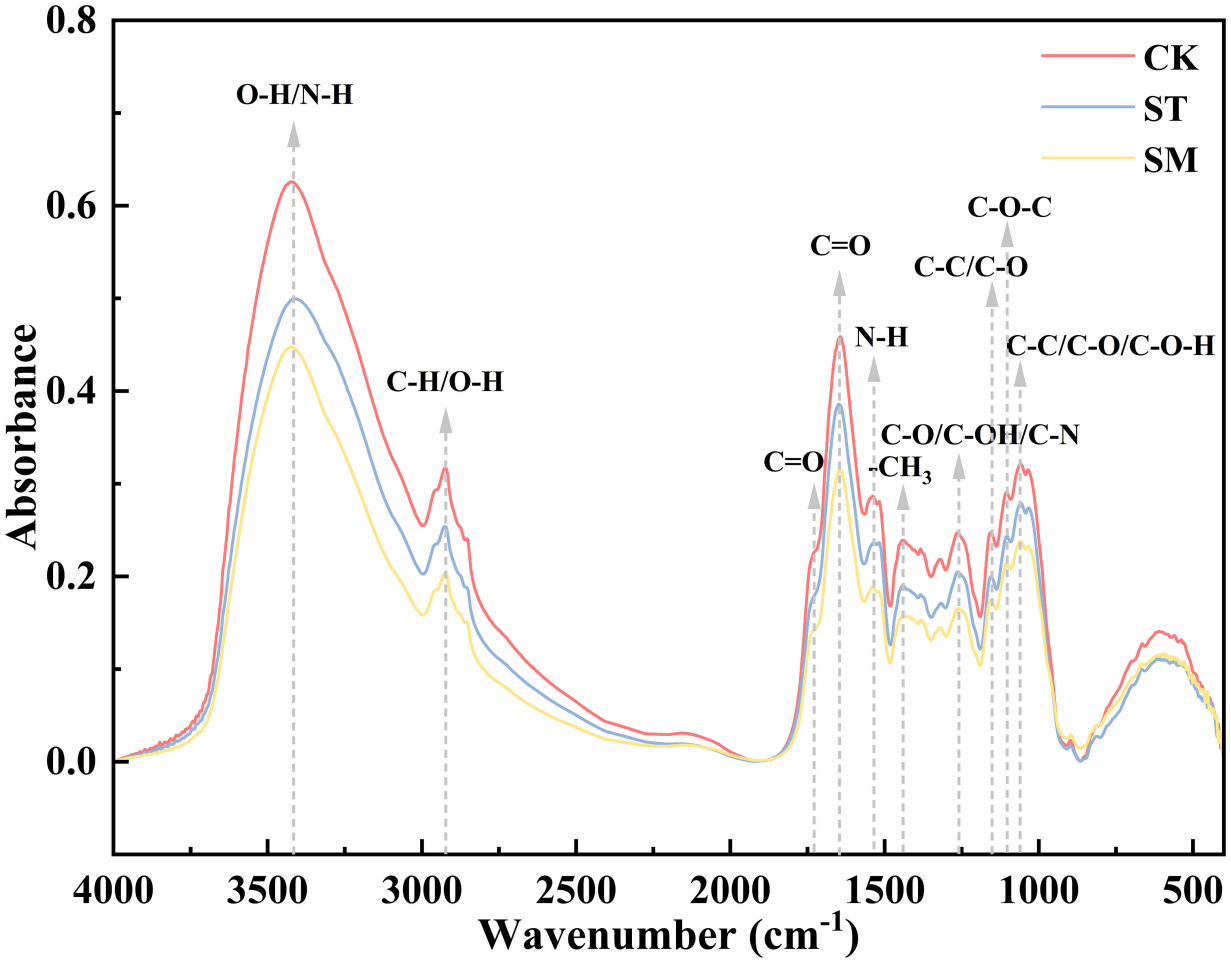
FTIR spectra of root cell walls from *Perilla frutescens* plants under different treatments.

**Figure 4** presents the infrared spectra of root cell walls under three different treatments, with **Table 1** listing the main absorption peaks in the 4000–400 cm^-1^ region. Compared to the control group, the absorption peaks under Cd stress were lower and narrower, and this effect was further intensified with the addition of microalgae. However, the overall spectral profiles remained largely unchanged across treatments, indicating that the fundamental structure of the root cell walls was not substantially altered. Nonetheless, shifts in specific absorption peaks reflect changes in the composition and conformation of functional groups. Specifically, a broad and intense absorption band at ~3420 cm^−1^, corresponding to the O-H in cellulose and pectin or N-H of amines, shifted from 3419.84 cm^−1^ (CK) to 3408.49 cm^−1^ (ST) under Cd stress, and further to 3423.61 cm^−1^ (SM) with microalgae. The peak near 1538 cm^−1^, associated with N-H bending vibrations of amide II, shifted from 1538.92 cm^−1^ to 1534.42 cm^−1^ under Cd, and to 1540.89 cm^−1^ with microalgae. These changes point to the involvement of polysaccharide and protein groups in Cd adsorption, with microalgae influencing their vibrational behavior. Peaks related to C-H vibrations in lignin and pectin (2924–2925 cm^−1^) and C=O stretching in amide I (1643–1648 cm^−1^) exhibited upward shifts in frequency under Cd stress. In contrast, the C=O band around 1730 cm^−1^, linked to esterified pectin or carbonyl groups, showed a Cd-induced decrease in frequency. The absorption bands within 1058–1156 cm^−1^, corresponding to C-C, C-OH, C-O-C, or C-O-H vibrations of polysaccharides, also shifted to lower frequencies under Cd stress. However, these changes were partially reversed by microalgae treatment. Moreover, the -CH_3_ bending vibration at 1440 cm^−1^ and the C-O/C-OH or C-N stretching band at 1262 cm^−1^ shifted to lower frequencies under Cd and were further displaced following microalgae treatment, indicating that microalgae may reinforce Cd-induced conformation changes in certain cell wall components.

**Table 1:** FTIR spectra characterization of root cell wall components in *Perilla frutescens* plants across different treatments.

Functional group / Wavenumber (cm-1)	CK	ST	SM
O-H stretching of hydroxyl groups, N-H of amines	3419.84	3408.49	3423.61
C-H stretching of CH_3_, O-H of carboxyl groups	2924.29	2925.58	2925.17
C=O stretching vibration of esters or carbonyl groups	1730.32	1724.53	1735.14
C=O stretching in amide I	1643.46	1648.07	1644.40
N-H bending of amide II	1538.92	1534.42	1540.89
-CH_3_ bending of polysaccharides	1442.46	1440.39	1437.85
C-O or C-OH stretching of polysaccharides, C-N stretching of amide III	1263.05	1262.41	1260.44
C-C or C-O stretching of polysaccharides	1156.12	1155.15	1156.12
C-O-C stretching in polysaccharides	1104.53	1104.05	1104.53
C-C, C-O or C-O-H stretching of polysaccharides	1059.27	1058.71	1058.86

The FTIR analysis further supported the role of microalgae in promoting root cell wall remodeling under Cd stress. Previous studies have indicated that oxygen-containing functional groups in cell wall polysaccharides, such as -COOH and -OH, serve as primary binding sites for heavy metals (Lin et al., 2009; Meychik et al., 2021; Yu et al., 2021). The quantity and chemical state of these functional groups significantly influence the ability of plant cell wall to bind divalent metal cations (Meychik et al., 2021). In this study, microalgae treatment induced marked shifts in functional groups associated with Cd binding, including OH from pectin, CH_3_ and C=O from pectin and hemicelluloses, and C-C or C-O-H from cellulose and hemicelluloses. These spectral changes reflected conformational rearrangements in pectin, cellulose and hemicelluloses, which likely optimized the spatial orientation and chemical reactivity of metal-binding groups, thereby facilitating Cd immobilization in the cell wall (Wei et al., 2023; Li A. et al., 2024). Notably, when microalgae treatment was applied, the Cd-binding capacity of hemicellulose I significantly increased despite no observable change in its content, pointing to potential structural modifications. The FTIR-detected vibrational shifts of functional groups such as C-C, C-O, C-O-H, and C=O under microalgae treatment partially support this inference. These spectral changes may reflect alterations in hydrogen bonding interactions, backbone conformation, side chain composition, or the degree of esterification of hemicellulose I, possibly increasing the exposure or spatial availability of functional groups such as hydroxyl and carboxyl groups involved in Cd coordination. Additionally, shifts in peaks associated with structural proteins implied potential adjustments of metal-binding domains, further supporting enhanced Cd sequestration in root tissues (Lin et al., 2009).

### 3.8 Root exudate metabolomic profiling

#### 3.8.1 Metabolite identification

A total of 2,083 compounds were identified in the root exudates of *Perilla frutescens*, with 1,363 detected in positive ion mode and 720 in negative ion mode (**Supplementary Table S6**). PCA showed clear separations among the CK, ST, and SM groups, indicating significant alterations in root exudate profiles in response to Cd stress and microalgae treatment (**Supplementary Figure S6A**). To further explore these metabolic changes, PLS-DA was conducted to identify DAMs (**Supplementary Figure S6C, D**), revealing 869 DAMs (384 upregulated/485 downregulated) for ST vs CK comparison and 534 DAMs (276 upregulated/258 downregulated) for SM vs ST comparison (**Supplementary Figure S6E, F**). According to HMDB annotations, these DAMs were primarily classified as lipids and lipid-like molecules, phenylpropanoids and polyketides, organoheterocyclic compounds, and organic acids and derivatives (**Supplementary Figure S6B**).

Under Cd stress, specific compounds were significantly upregulated, notably lysine butyrate, along with various phenylpropanoids and polyketides, while downregulated metabolites were predominantly lipids and lipid-like molecules (**Supplementary Table S6**, **Supplementary Figure S7**). Compared with the Cd-only group, microalgae treatment induced a greater number of upregulated metabolites, especially organic acids—mainly amino acids, peptides, and analogues—and organoheterocyclic compounds. Camptothecin and pyridoxamine 5-phosphate exhibited the highest fold changes. On the other hand, lipids and lipid-like molecules continued to dominate the downregulated metabolites, with phosphocholine showing the greatest reduction.

Root exudates are actively involved in metal mobilization, complexation, and detoxification (Chen et al., 2017). In addition to directly modulating heavy metal bioavailability via acidification, chelation, precipitation, and redox processes, they can also indirectly affect metal uptake by regulating root development, altering rhizosphere conditions, and shaping plant-microbe interactions (Bali et al., 2020). Among these, low-molecular-weight organic acids such as tartaric, oxalic, malic, and citric acids are well known to enhance Cd solubilization and facilitate its translocation to aboveground tissues (Agarwal et al., 2024; Zhang et al., 2024). These effects are largely attributed to the formation of soluble Cd-organic acid complexes. For example, malic acid, tartaric acid, and oxalic acid secreted by *Sedum alfredii* roots can form stable complexes with Cd, promoting its mobilization in the rhizosphere (Tao et al., 2016; Tao et al., 2020b), while Liao et al. (2023) demonstrated that oxalic, malic, and citric acids enhanced Cd translocation via xylem transport in *Brassica juncea*. Similarly, exogenous application of citric acid and glutaric acid has been shown to promote Cd uptake and translocation in sunflower (Niu et al., 2023). Despite these documented roles, the present metabolomic analysis did not reveal a significant increase in their levels in the microalgae co-treatment group compared to the Cd-only treatment, suggesting that they may not be the primary contributors to the enhanced Cd accumulation observed in this case. Instead, microalgae addition was associated with elevated levels of amino acid-related metabolites, such as L-aspartic acid, L-threonine, L-homocystine, tyrosylalanine, and valylproline. These molecules contain functional groups (e.g., amino and carboxyl groups) capable of forming stable coordination complexes with Cd and may contribute to Cd chelation or translocation. Aspartic acid and threonine have been reported to enhance Cd uptake in Chinese cabbage when applied exogenously (Li L. et al., 2024). Moreover, amino acids play important roles in maintaining cellular osmotic homeostasis and redox balance. Their accumulation can protect cells from oxidative damage and facilitate Cd detoxification (Sun et al., 2020). Increased levels of tyrosine derivatives observed here may indicate enhanced synthesis of phenolic compounds, which support antioxidant defenses and metal chelation in the rhizosphere (Guo et al., 2016; Bali et al., 2020). Plant hormones are also implicated in Cd stress adaptation. Notably, methyl jasmonate (MeJA) and dihydrozeatin were also significantly elevated in the microalgae-treated group. MeJA has been reported to improve Cd tolerance in various species, such as rice, cucumber, and *Cosmos bipinnatus*, by enhancing biomass production, improving nutrient uptake, stabilizing photosynthetic function, regulating cell structure and boosting ROS-scavenging systems (Kanu et al., 2022; Fan et al., 2024; Niu et al., 2025). Dihydrozeatin, a type of cytokinin, regulates cell differentiation and development and may help maintain root structure and function under stress conditions (Vinciarelli et al., 2025). Since a substantial proportion of photosynthetic products are released into the rhizosphere, metabolic profiles of root exudates can reveal how microalgae influence the adaptive responses of *Perilla frutescens* to Cd stress (Feng et al., 2021). The enrichment of vitamin B6 derivatives, particularly pyridoxamine 5-phosphate, could reinforce the antioxidant defense, while the upregulation of camptothecin suggests possible activation of signaling pathways related to stress response or secondary metabolism (Lu et al., 2022; Yeshi et al., 2022). In contrast, downregulation of lipids, particularly the marked decrease in phosphocholine, may reflect membrane remodeling or alterations in lipid-mediated stress signaling (Laureano et al., 2025; Oubohssaine et al., 2025).

#### 3.8.2 Key marker metabolites identification

**Figure 5:**
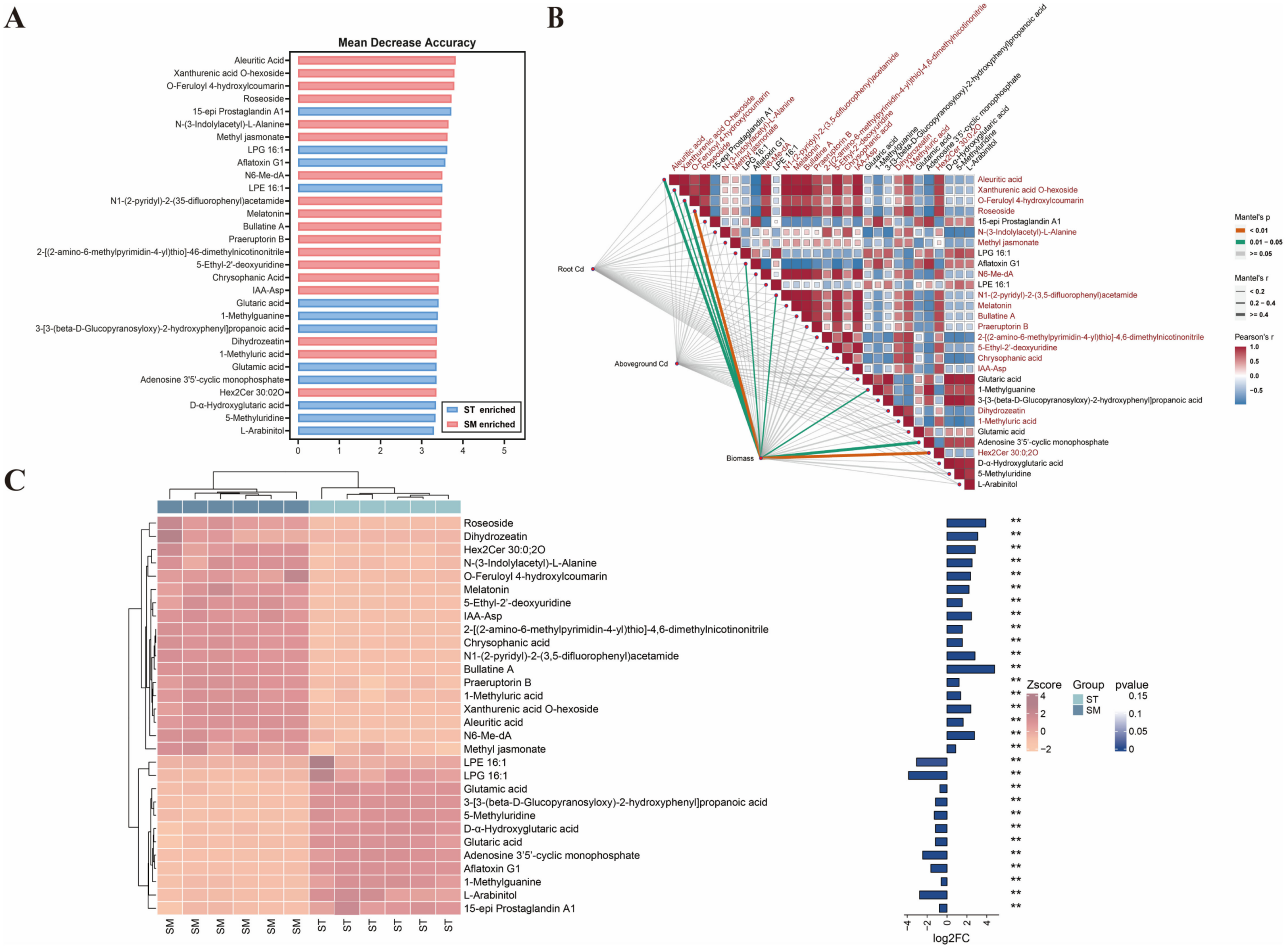
The random forest analysis of differentially accumulated metabolites (DAMs) in root exudates between the Cd/microalgae co-treated group (SM) and Cd-only treated group (ST). **(A)** Top 30 marker metabolites identified by the random forest classification, ranked by their contribution to model accuracy and stability. **(B)** Heatmap showing metabolite correlations, accompanied by Mantel analysis demonstrating the relationships among metabolites, Cd concentrations in plant tissues, and plant biomass. Metabolites enriched in the SM group are highlighted in red. **(C)** Cluster heatmap showing the differential abundances of metabolites between the SM and ST groups. The bar plot on the right shows log_2_ fold change (log_2_FC) values and asterisks indicate statistical significance based on *p*-values. **p<0.01.

To pinpoint key metabolites contributing to microalgae-enhanced Cd accumulation and tolerance, a random forest analysis was applied to the identified DAMs. The top 30 metabolites contributing most to the model accuracy and stability were identified (**Figure 5**). Among these, 18 metabolites were significantly enriched in the SM group compared to ST. However, Mantel analysis revealed no significant correlation between these DAMs and Cd concentrations in plant tissues. Despite this, nine of these metabolites were positively correlated with plant biomass (*p*<0.05), implying that their functions are more likely associated with enhancing Cd tolerance than directly promoting Cd uptake.

**Figure 6:**
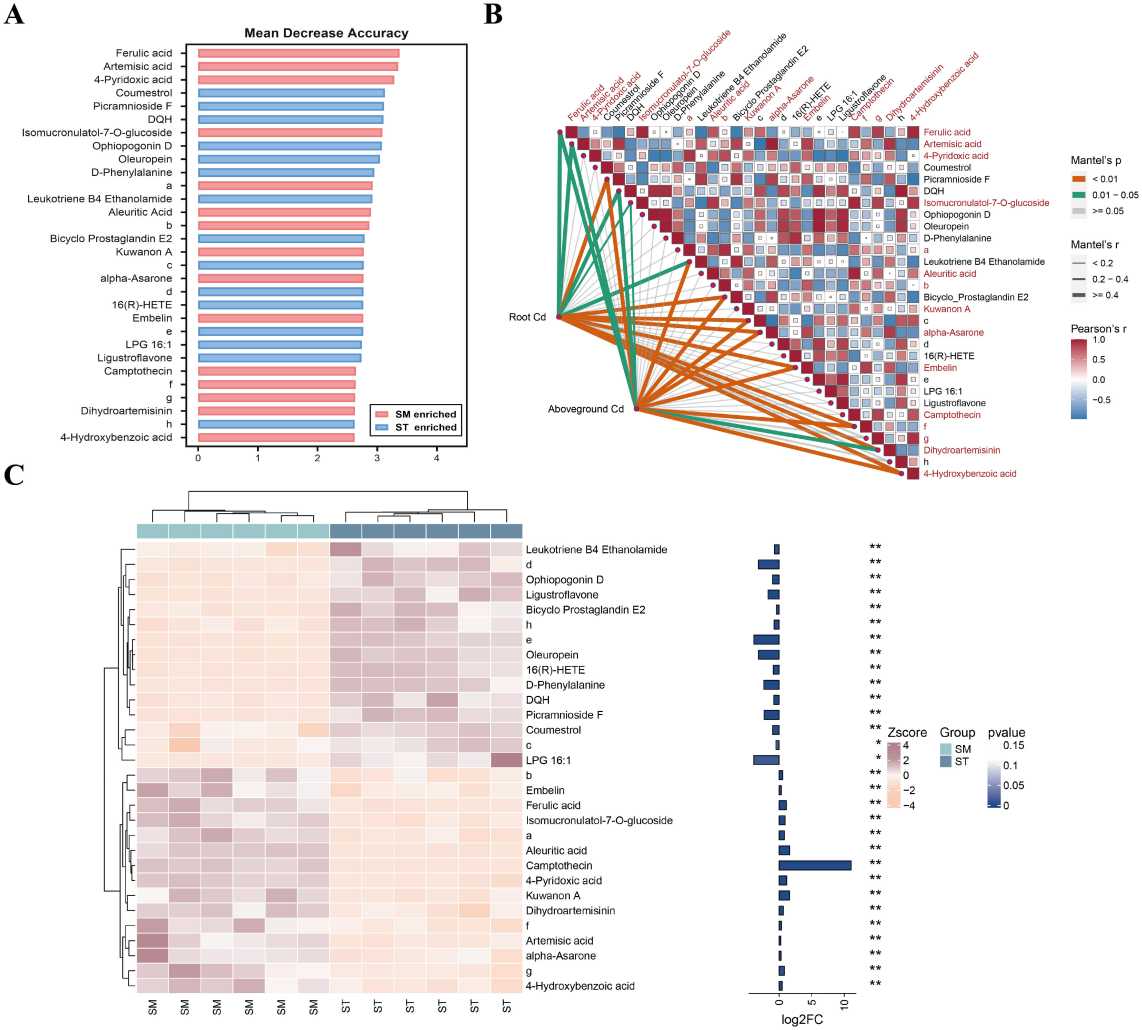
The random forest analysis of all detected metabolites in the root exudates of Cd/microalgae co-treated (SM) and Cd-only treated (ST) samples. **(A)** Top 30 marker metabolites identified by the random forest classification, ranked by their contribution to model accuracy and stability. **(B)** Heatmap showing metabolite correlations, accompanied by Mantel analysis demonstrating the relationship between metabolites and Cd concentrations in plant tissues. Metabolites enriched in the SM group are highlighted in red. **(C)** Cluster heatmap showing the differential abundances of metabolites between the SM and ST groups. The bar plot on the right shows log_2_ fold change (log_2_FC) values and asterisks indicate statistical significance based on *p*-values. *p<0.05, **p<0.01. Note: a, 4-(beta-D-Glucopyranosyloxy)-2-methylenebutanoic acid; b, 5-(6-hydroxy-6-methyloctyl)-2,5-dihydrofuran-2-one; c, 2,6-di[2-chloro-4-(dimethylamino)benzylidene]cyclohexan-1-one; d, methyl 1-isopropyl-1H-1,2,3-benzotriazole-5-carboxylate; e, 6,15-diketo-13,14-dihydro Prostaglandin F1α; f, (1S,2S)-Nicotine1-oxide; g, 2-(3,4-dihydroxyphenyl)-3,5,7-trihydroxy-6-methyl-4H-chromen-4-one; h, N-(1-benzyl-4-piperidinyl)-4-(1H-pyrazol-1-yl)benzamide.

Given this result, it was hypothesized that non-differentially accumulated metabolites may play a more important role in Cd accumulation. Therefore, the random forest analysis was expanded to include all detected metabolites, identifying 30 key metabolites differentiating the SM and ST groups (**Figure 6**). Among these, 15 metabolites, mainly phenolic acids and flavonoids, were significantly enriched in the SM group. Eight metabolites showed significant positive correlations with Cd accumulation: ferulic acid, artemisic acid, isomucronulatol-7-O-glucoside, alpha-asarone, embelin, (1S,2S)-nicotine1-oxide, dihydroartemisinin, and 4-hydroxybenzoic acid. The findings suggested that these metabolites are likely functional contributors to the enhanced Cd accumulation observed under microalgae treatment.

The distinction between DAMs and non-DAMs reveals a functional divergence in how these metabolites respond to microalgae treatment. While DAMs are more closely linked to plant biomass, non-DAMs, especially phenolic acids and flavonoids, appear to play a more important role in Cd accumulation regulation and stress adaptation. For instance, phenolic compounds such as ferulic acid and 4-hydroxybenzoic acid are well-documented for their ability to chelate heavy metals through hydroxyl and carboxyl groups, and can form more stable complexes with Cd than low-molecular-weight organic acids (Bali et al., 2020). Such phenolic compounds not only facilitates Cd uptake and translocation within plants, but also contributes to ROS detoxification, thereby optimizing metal accumulation while alleviating cytotoxicity (Lanoue et al., 2010; Bali et al., 2020; Chen et al., 2020). Equally important are specialized metabolites like isomucronulatol-7-O-glucoside and embelin, whose potent antioxidant properties help stabilize cellular structures and scavenge stress-induced ROS (Caruso et al., 2020; Rao et al., 2022). Moreover, terpenoids enriched in microalgae-treated samples, such as artemisinin and dihydroartemisinin, have been shown in previous studies to possess antifungal and nematicidal activity, which may enhance plant resistance (You et al., 2021; Wang et al., 2022). These metabolites, though not meeting the differential metabolite screening criteria, may still be key regulators within the plant stress response network.

#### 3.8.3 KEGG pathway enrichment analysis

**Figure 7:**
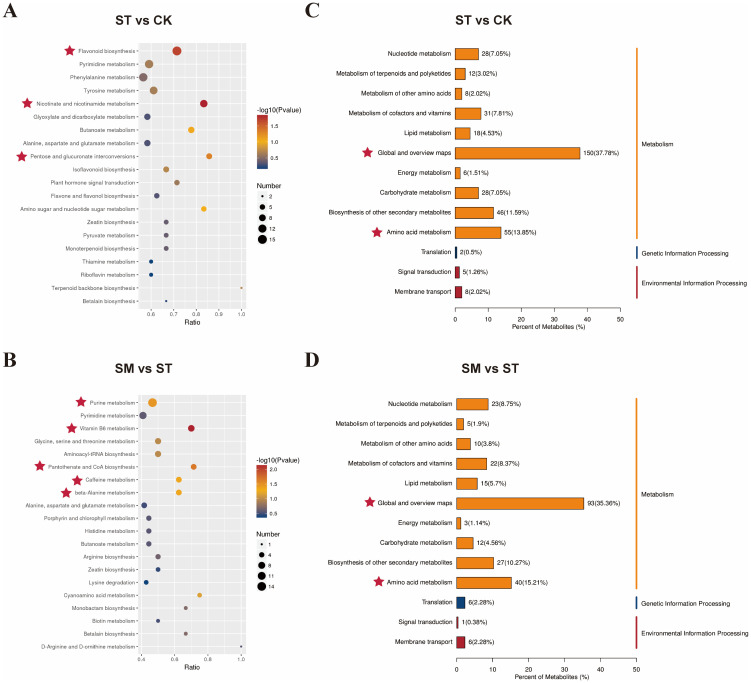
KEGG enrichment analysis of differentially accumulated metabolites (DAMs) from various treatment comparisons. **(A, B)** show bubble plots of the top 20 enriched KEGG pathways for DAMs from different comparisons. The x-axis represents the enrichment factor (the number of DAMs in a given pathway divided by the total number of identified metabolites in that pathway). Bubble color indicates the *p*-value from the hypergeometric test, and bubble size reflects the number of DAMs in each pathway. **(C, D)** present KEGG classification of DAMs. The x-axis represents the proportion of metabolites assigned to each KEGG pathway relative to the total number of annotated metabolites. The right y-axis shows the primary KEGG pathway classifications and the left y-axis displays the corresponding secondary classifications.

KEGG pathway enrichment analysis revealed that Cd and microalgae treatment primarily affected pathways related to global and overview maps and amino acid metabolism (**Figure 7C, D**). Compared to the control, Cd treatment significantly enriched DAMs involved in flavonoid biosynthesis, nicotinate and nicotinamide metabolism, and pentose and glucuronate interconversions (**Figure 7A**). The enrichment of flavonoid biosynthesis reflects a typical stress response, where upregulated metabolites like luteolin and kaempferol contribute to metal ions chelation and ROS scavenging (**Supplementary Table S6**) (Rice-Evans et al., 1996; Borowska et al., 2020; Jomova et al., 2023; Zhu et al., 2024). The activation of nicotinate and nicotinamide metabolism and pentose and glucuronate interconversions likely supports the biosynthesis of NAD^+^, NADP^+^, and NADPH, key cofactors in redox regulation and energy metabolism (Decros et al., 2019; Ahmad et al., 2021). These cofactors drive NADPH-dependent antioxidant defense, such as the glutathione cycle, which are critical for counteracting Cd-induced oxidative stress (Ishikawa et al., 2017; Xiao et al., 2020; Cassier-Chauvat et al., 2023; Rathore et al., 2024).

When microalgae were introduced, notable enrichment was observed in pathways including vitamin B6 metabolism, pantothenate and CoA biosynthesis, purine metabolism, caffeine metabolism and beta-alanine metabolism relative to Cd-only treatment (*p*<0.05) (**Figure 7B**). The induction of the pantothenate and CoA biosynthesis pathway implies heightened energy demands and improved stress adaptation, while the enrichment of vitamin B6 metabolism may contribute to enhanced antioxidant defenses and tolerance to heavy metal stress (Jonczyk et al., 2008; Mwamba et al., 2020; Wang et al., 2020; Song et al., 2024; Yan et al., 2024). Amino acid metabolism plays a crucial role in plant Cd adaptation and may influence Cd complexation and accumulation (Bali et al., 2020). In particular, alanine metabolism is considered a key pathway associated with Cd tolerance (Sun et al., 2020; Saeed et al., 2024). The caffeine metabolism pathway primarily involves the methylation and degradation of xanthine derivatives, which may help modulate defense capabilities of plants (Xu et al., 2024). In short, these observations reflected that microalgae supplementation enhanced plant resistance to Cd stress through coordinated improvements in antioxidant capacity, energy metabolism, and cellular repair processes.

### 3.9 Interconnected root adaptations driving microalgae-enhanced Cd uptake

Based on the integrative analysis of physiological, structural, and biochemical responses, a mechanistic model is proposed to illustrate how microalgae promote Cd accumulation in *Perilla frutescens* through a dynamic and coordinated regulation at the root level. Firstly, microalgae treatment enhanced root antioxidant capacity, notably through increased POD activity, which reduced ROS accumulation and alleviated oxidative stress. This enhanced antioxidant defense helps maintain cellular homeostasis and metabolic activity, thereby preserving root physiological vigor and enabling efficient Cd uptake. Concurrently, structural modifications in the root cell wall, particularly in the hemicellulose I fraction, increase Cd retention capability. Such remodeling not only contributes to the immobilization of Cd within root tissues but also helps maintain structural integrity and functional stability under heavy metal stress. In parallel, microalgae treatment altered the composition and abundance of root exudates, promoting the release of compounds with metal-chelating, antioxidant, and plant growth-promoting properties. These exudates enhanced Cd bioavailability in the rhizosphere, while also contributing to root protection through ROS scavenging. Additionally, exudate-driven modulation of the rhizosphere microbial community may facilitate the recruitment of beneficial microbes that promote plant nutrient acquisition and stress tolerance (Upadhyay et al., 2022).

These mechanisms act synergistically to constitute an integrated adaptive strategy. Enhanced antioxidant activity maintains cellular homeostasis and supports a metabolically active root state, which facilitates the secretion of functional exudates. These exudates, in turn, contribute to maintaining root physiological activity and structural development by modulating the rhizosphere environment. Meanwhile, structural regulation of root cells reinforces root stability and sustains root function under stress, enabling the root system to maintain physiological activity and exudation. Together, these processes form a microalgae-driven positive feedback loop that amplifies Cd accumulation while preserving plant vitality under stress conditions.

## 4 Conclusion

The application of microalgae enhanced Cd bioaccumulation in *Perilla frutescens*, attributed to improved Cd uptake efficiency and greater plant biomass. This enhancement was closely associated with microalgae-induced modifications in root physiology and structure, including elevated antioxidant responses, remodeling of the cell wall, and changes in root exudate profiles. Microalgae treatment increased POD activity in roots, alleviated lipid peroxidation and suppressed ROS generation. Also, it markedly promoted the secretion of defense-related compounds, particularly amino acid-related metabolites, phenolic acids, and flavonoids, which likely improved Cd bioavailability and plant tolerance through metal-binding and antioxidant properties. Furthermore, microalgae treatment mitigated the subcellular damage in root apical region and reshaped Cd partitioning within the root cell wall, significantly increasing Cd retention in hemicellulose I. These effects were accompanied by shifts in the absorption characteristics of functional groups related to Cd binding, reflecting improved structural stability and enhanced phytoextraction efficiency. Although this study was conducted under hydroponic conditions, the findings highlight the potential of microalgae as an effective tool for improving phytoremediation outcomes. This research offers theoretical support for improving agricultural and environmental management, particularly in the development of sustainable bioremediation strategies for heavy metal-polluted soils. However, further evaluations are needed to assess the scalability of microalgae application in field environments.

## Data Availability

The original contributions presented in the study are included in the article/**Supplementary Material**. Further inquiries can be directed to the corresponding authors.
