# Supplementary material for: Microalgae enhance cadmium accumulation in Perilla frutescens: root structural adaptation and secretion-mediated detoxification
Source: Front Plant Sci. 2025 Sep 2;16:1642230. doi: 10.3389/fpls.2025.1642230 (PMC12436388; doi:10.3389/fpls.2025.1642230)
Supplement: Supplementary file 1 [file DataSheet1.docx]

**Microalgae enhance cadmium accumulation in *Perilla frutescens*: Root structural adaptation and secretion-mediated detoxification**

Ying Ren, Yuying Su, Jinfeng Li, Hui Zhang, Yumeng Yang, Yuanyuan Li, Juan Chen, Xiaohui Pang, Zheng Zhang^*^, Jianping Han^*^

*Institute of Medicinal Plant Development,* *Chinese Academy of Medical Sciences & Peking Union Medical College, Beijing 100193, China*

^*^Corresponding author: Jianping Han & Zheng Zhang

E-mail: happymyra2007@163.com; zhangzheng321@126.com

**Supplementary materials**

**Table S1**. Composition of Hoagland’s modified nutrient solution used in the study.

| **Component** | **Concentration (mg/L)** |
| --- | --- |
| KNO_3_ | 506 |
| NH_4_NO_3_ | 80 |
| KH_2_PO_4_ | 136 |
| MgSO_4_ | 241 |
| FeNaEDTA | 36.7 |
| KI | 0.83 |
| H_3_BO_3_ | 6.2 |
| MnSO_4_ | 22.3 |
| ZnSO_4_ | 8.6 |
| Na_2_MoO_4_ | 0.25 |
| CuSO_4_ | 0.025 |
| CoCl_2_ | 0.025 |
| Ca(NO_3_)_2_ | 945 |

**Table S2.** The parameters of Q Exactive™ HF-X mass spectrometer.

| **Items** | **Value** |
| --- | --- |
| Spray voltage | 3.5 kV |
| Capillary temperature | 320℃ |
| Sheath gas flow rate | 35 psi |
| Aux gas flow rate | 10 L/min |
| S-lens RF level | 60 |
| Aux gas heater temperature | 350℃ |

**Table S3.** The working parameters of apparatus.

| **ICP-MS Parameter** | **Value** |
| --- | --- |
| RF power | 1550 W |
| Pump Speed | 40 rpm |
| Cool flow | 14 L/min |
| Auxilliary flow | 0.8 L/min |
| Nebulizer flow | 1.00 L/min |

**Table S4.** Details of the assay kits used for evaluating root antioxidant activity.

| **Indicator** | **Manufacturer** | **Method** |
| --- | --- | --- |
| Superoxide anion radical content / production rate | Shanghai yuanye Biotechnology Co., Ltd | By measuring the absorbance of a pink azo compound formed from NO_2_^-^, which is produced by the reaction of O_2_^-^ with hydroxylamine, reacting with p-aminobenzenesulfonic acid and naphthylamine under acidic conditions. |
| SOD activity | Beijing Solarbio Science & Technology Co.,Ltd. | WST-1 method. |
| POD activity | Beijing Solarbio Science & Technology Co.,Ltd. | By measuring the absorbance of the brown compound formed by the oxidation of guaiacol in the presence of hydrogen peroxide. |
| MDA content | Beijing Solarbio Science & Technology Co.,Ltd. | By measuring the absorbance of the reddish-brown compound formed from the reaction of MDA with thiobarbituric acid under acidic and high-temperature conditions. |

**Table S5.** Effect of different treatments on translocation factors (TFs) of *Perilla frutescens* plants.

| Treatment | TF *_root-to-stem_* | TF *_root-to-leaf_* | TF *_stem-to-leaf_* |
| --- | --- | --- | --- |
| ST | 0.464 ± 0.230a | 0.061 ± 0.031a | 0.151 ± 0.084a |
| SM | 0.498 ± 0.054a | 0.093 ± 0.042a | 0.184 ± 0.073a |

The values are presented as mean ± SD (standard deviation). The different lowercase letters indicate significant differences at *p* <0.05.


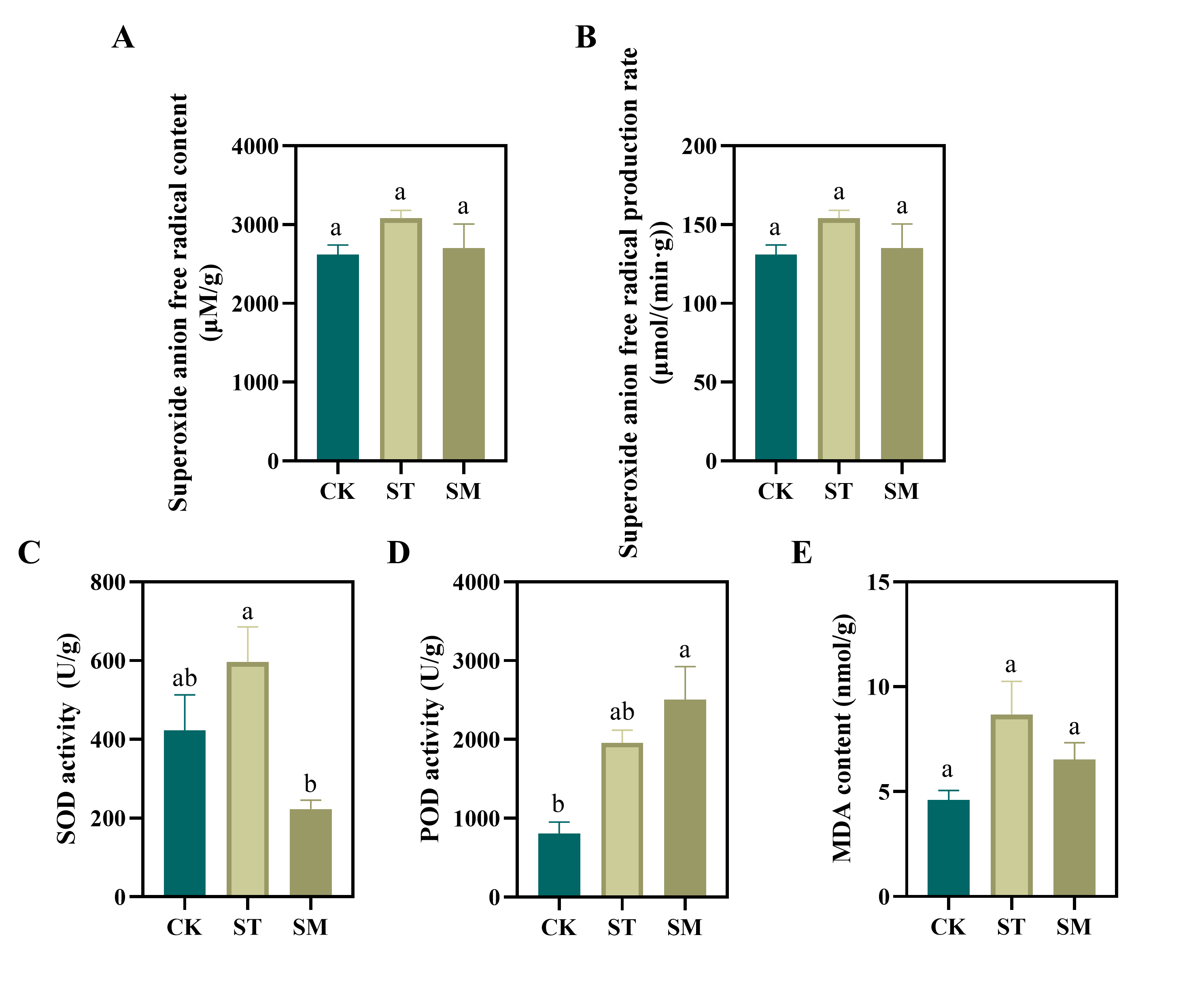


**Figure S1.** Effect of different treatments on superoxide anion free radical content (A), superoxide anion free radical production rate (B), SOD activity (C), POD activity (D), and MDA content (E) of *Perilla frutescens* roots. Note: The values are presented as mean ± SD (standard deviation). The different lowercase letters indicate significant differences at *p <*0.05.


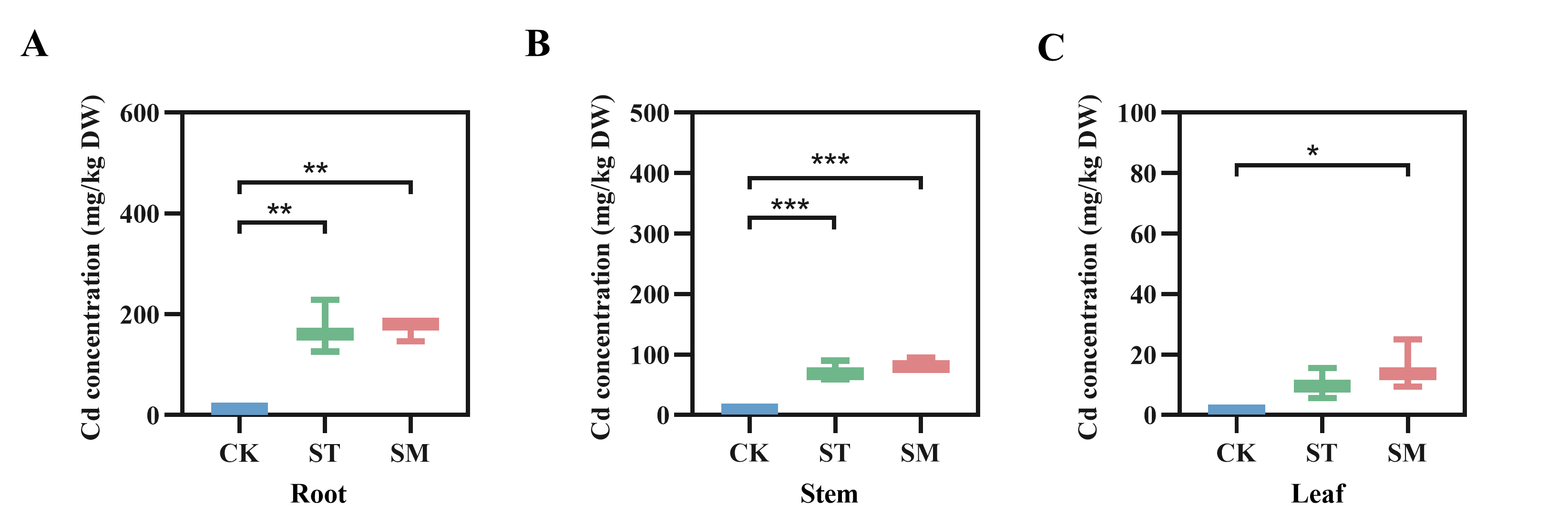


**Figure S2**. Effect of different treatments on Cd concentrations of *Perilla frutescens* roots (A), stems (B), and leaves (C). Note: The box plots display the median and interquartile range (IQR). Whiskers represent the minimum and maximum values within 1.5 times the IQR. *, *p* <0.05, **, *p* <0.01, ***, *p* <0.001.

**
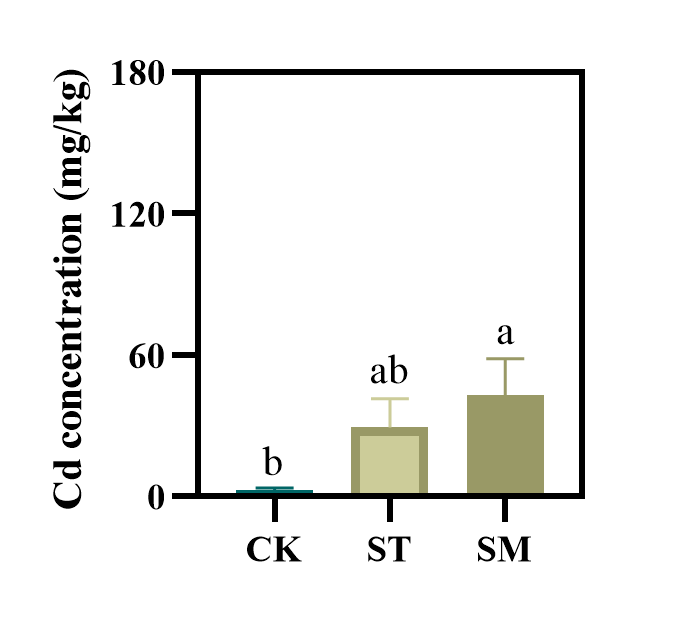
**

**Figure S3**. Cd concentrations in the root cell walls under different treatments. Note: The values are presented as mean ± SD (standard deviation). The different lowercase letters indicate significant differences at *p <*0.05.


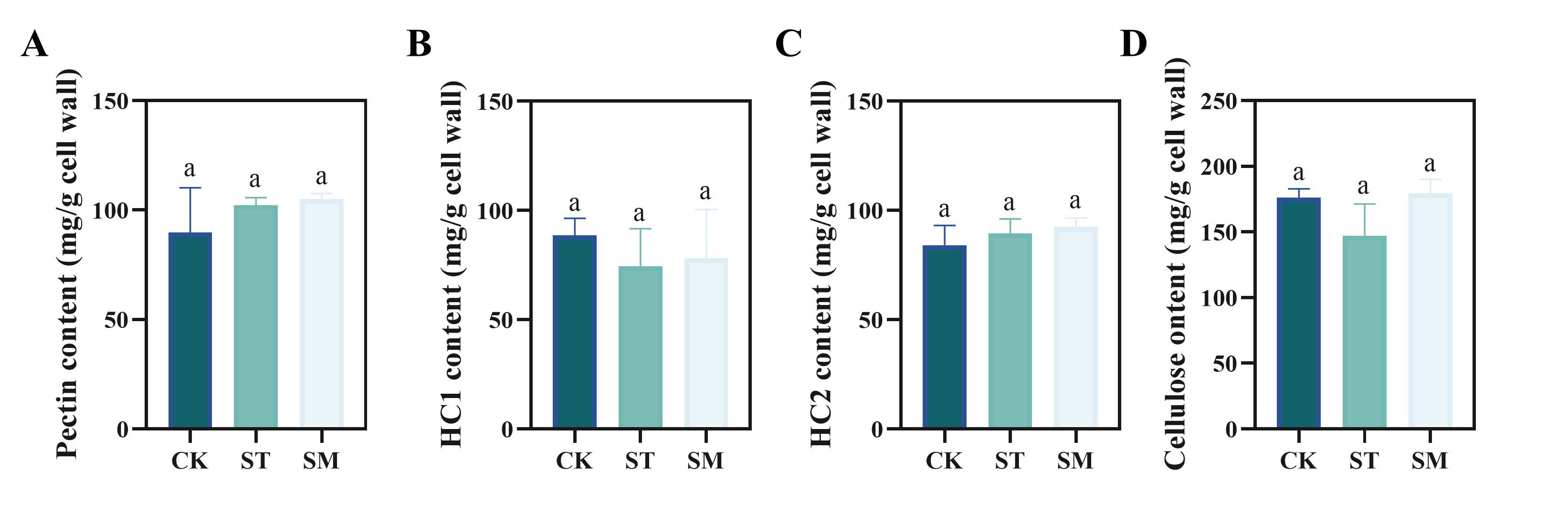


**Figure S4**. Effect of different treatments on the contents of pectin (A), hemicellulose Ⅰ (HC1) (B), hemicellulose Ⅱ (HC2) (C), and cellulose (D) in the root cell walls. Note: The values are presented as mean ± SD (standard deviation). The different lowercase letters indicate significant differences at *p <*0.05.


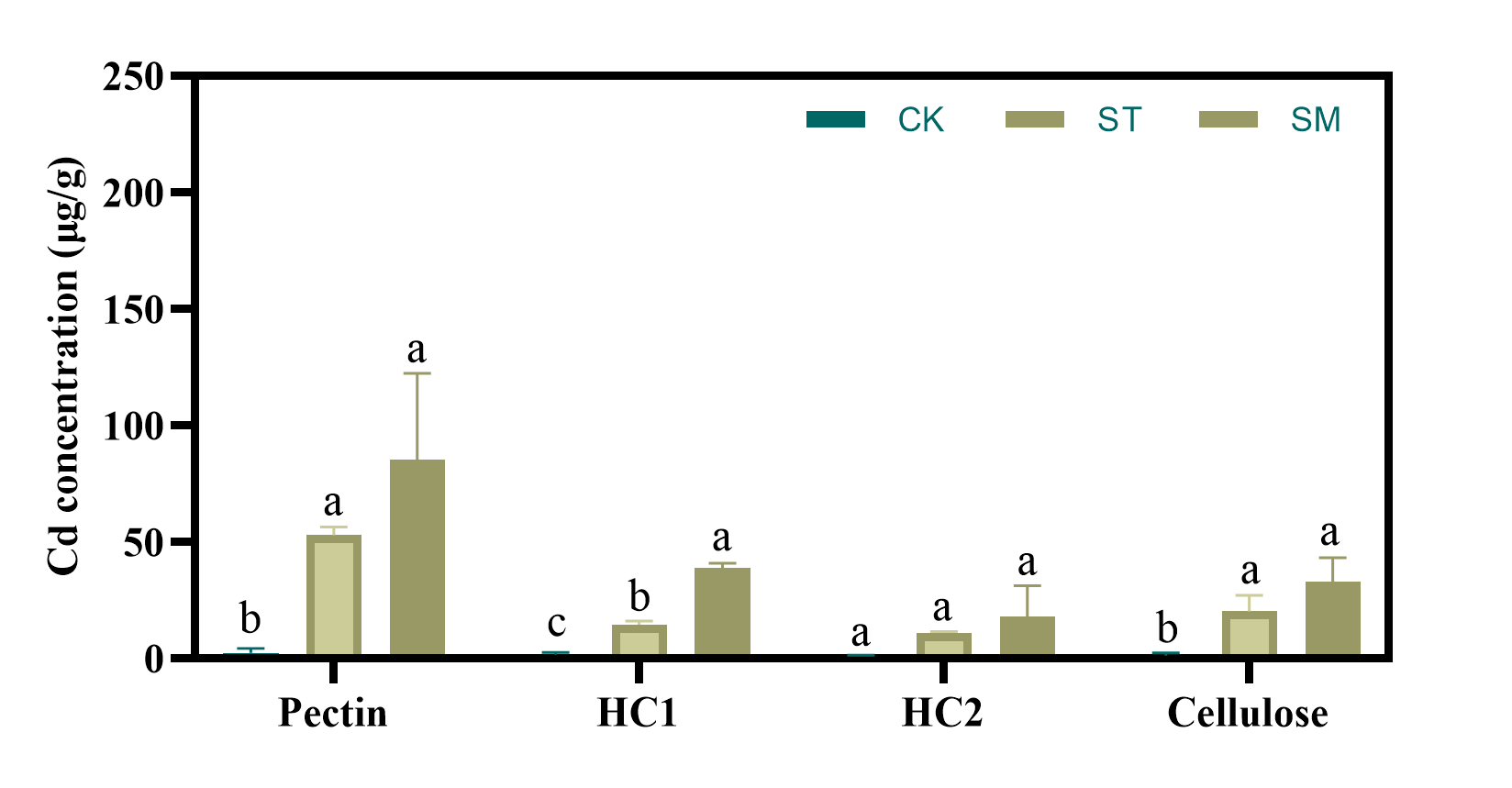


**Figure S5**. Cd concentrations in polysaccharide components of the root cell walls under different treatments. Note: The values are presented as mean ± SD (standard deviation). The different lowercase letters indicate significant differences at *p <*0.05.


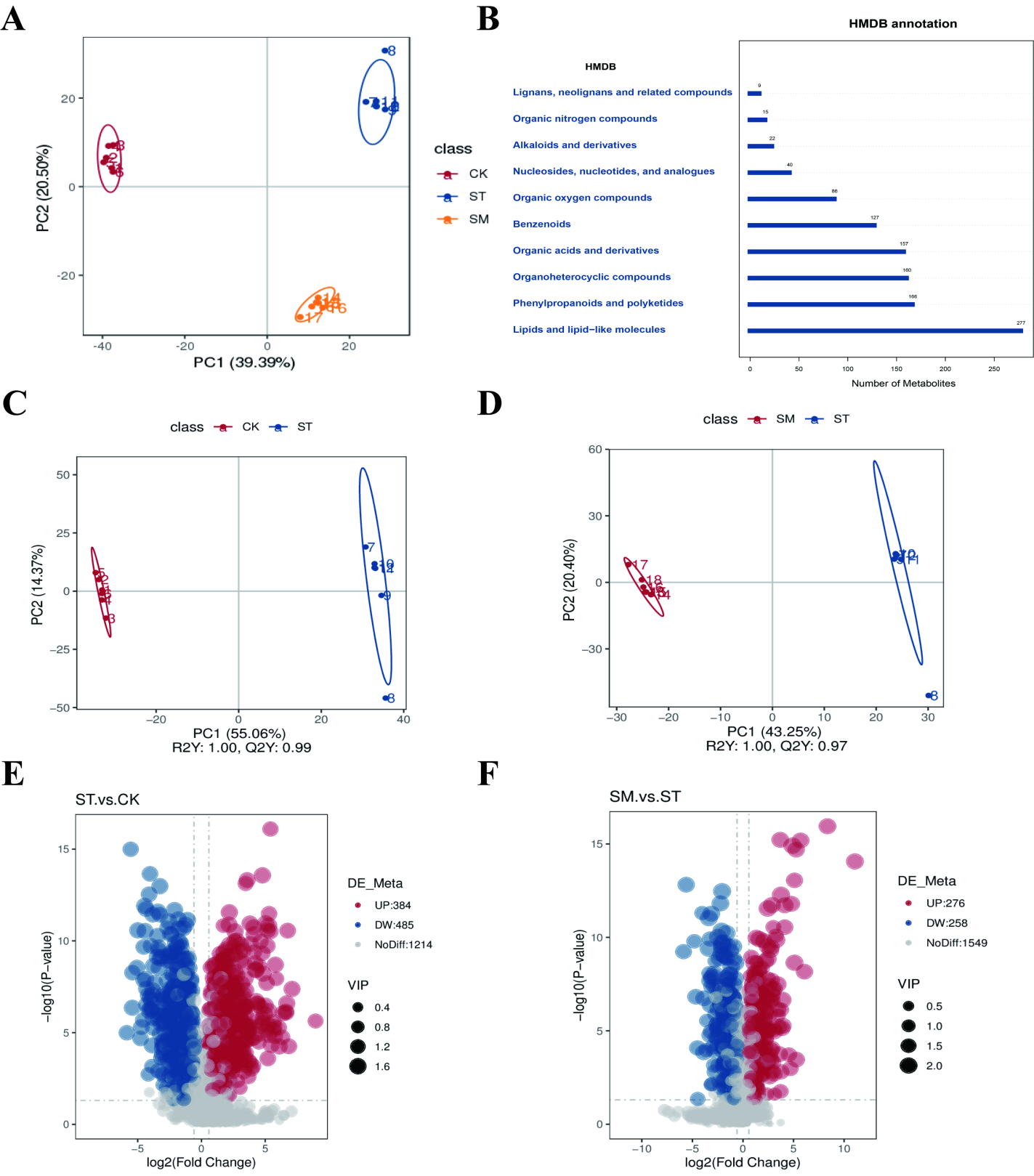


**Figure S6.** (A) Principal component analysis (PCA) of root exudate metabolites across different treatments. (B) Annotation of root exudate metabolites based on the HMDB. (C) Partial least squares discriminant analysis (PLS-DA) score plot comparing ST and CK groups. (D) PLS-DA score plot comparing SM and ST groups. (E) Volcano plots of differentially accumulated metabolites (DAMs) in ST vs CK comparison, with significance thresholds indicated for fold change and *p*-values. (E) Volcano plots of DAMs in SM vs ST comparison.


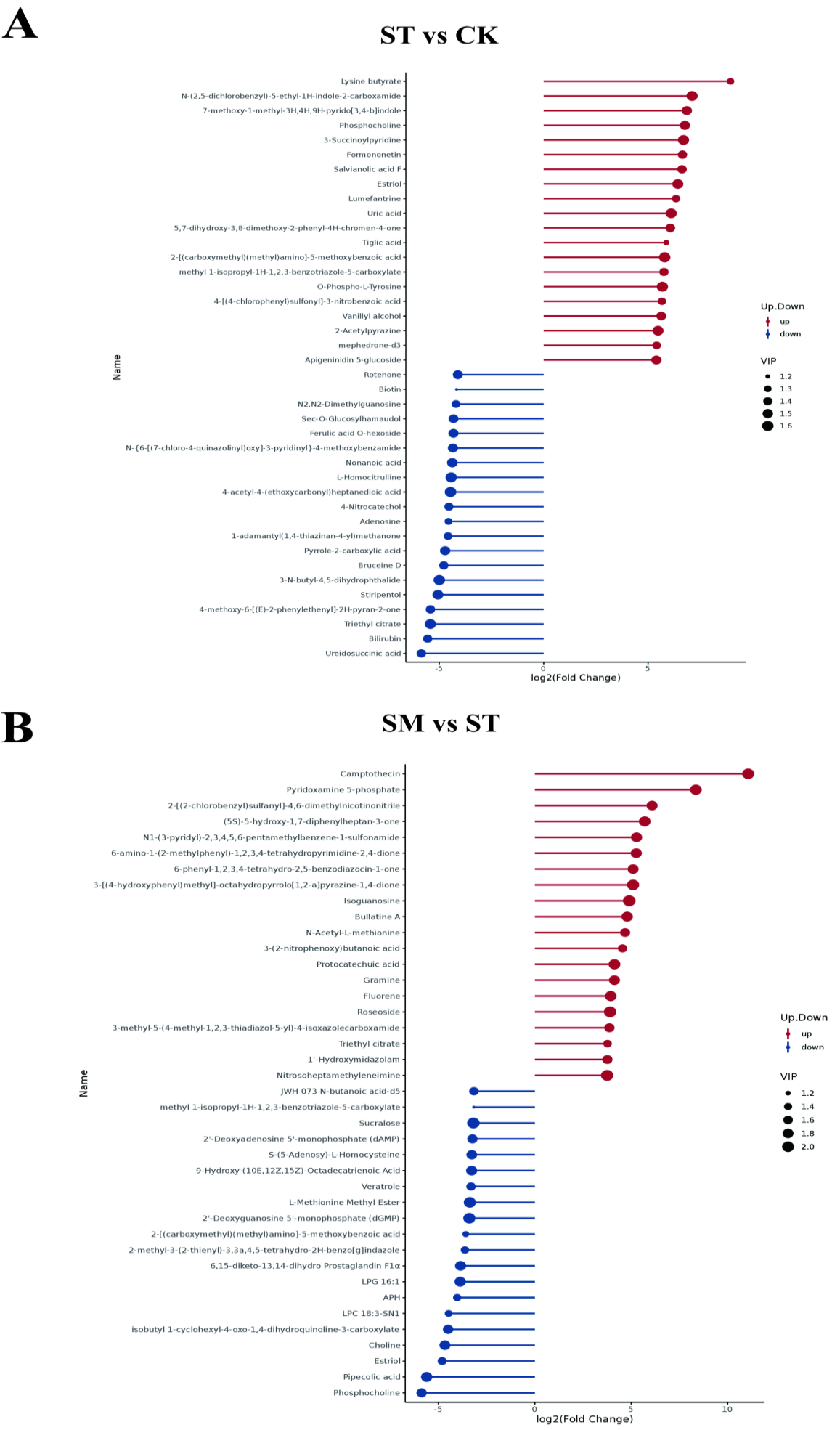


**Figure S7**. Top 20 differentially accumulated metabolites (DAMs) from different comparisons. The metabolites were ranked based on log_2_FC values, with red indicating up-regulation and blue indicating down-regulation. The stem length represents the log_2_FC magnitude, and the point size reflects VIP scores.
